# Long-term corticosteroid-induced chronic glaucoma model produced by intracameral injection of dexamethasone-loaded PLGA microspheres

**DOI:** 10.1080/10717544.2021.1998245

**Published:** 2021-11-12

**Authors:** MJ Rodrigo, D Garcia-Herranz, A Aragón-Navas, M Subias, T Martinez-Rincón, S Mendez-Martínez, MJ Cardiel, J García-Feijoo, J Ruberte, R Herrero-Vanrell, L Pablo, E Garcia-Martin, I Bravo-Osuna

**Affiliations:** a Department of Ophthalmology, Miguel Servet University Hospital, Zaragoza, Spain; b Miguel Servet Ophthalmology Research Group (GIMSO), Aragon Health Research Institute (IIS Aragon), Zaragoza, Spain; c National Ocular Pathology Network (OFTARED), Carlos III Health Institute, Madrid, Spain; d Complutense University of Madrid. Innovation, Therapy and Pharmaceutical Development in Ophthalmology (InnOftal) Research Group, UCM 920415, Department of Pharmaceutics and Food Technology, Faculty of Pharmacy, Complutense University of Madrid, Spain; e Health Research Institute, San Carlos Clinical Hospital (IdISSC), Madrid, Spain; f Miguel Servet Ophthalmology Research Group (GIMSO), University of Zaragoza, Aragon Health Research Institute (IIS Aragon), Zaragoza, Spain; g Department of Pathology, Lozano Blesa University Hospital, Zaragoza, Spain; h Complutense University of Madrid. Innovation, Therapy and Pharmaceutical Development in Ophthalmology (InnOftal) Research Group, UCM 920415. National Ocular Pathology Network (OFTARED), Carlos III Health Institute, Spain; i Servicio de Oftalmología, Hospital Clínico San Carlos, Madrid, Spain; j Departamento de Inmunología, Oftalmología y ORL, Facultad de Medicina, Universidad Complutense de Madrid (UCM), IdISSC, Madrid, Spain; k Animal Biotechnology and Gene Therapy Centre (CBATEG), Universitat Autònoma de Barcelona, Bellaterra, Spain; l Networked Biomedical Research Centre for Diabetes and Associated Metabolic Diseases (CIBERDEM), Madrid, Spain; m Department of Animal Health and Anatomy, School of Veterinary Medicine, Universitat Autònoma de Barcelona, Bellaterra, Spain

**Keywords:** Glaucoma, animal model, PLGA microspheres, neurodegeneration, dexamethasone

## Abstract

**Purpose:**

To evaluate a new chronic glaucoma model produced by intracameral injection of dexamethasone-loaded poly lactic-co-glycolic acid microspheres (Dex-PLGA-Ms) over six months.

**Methods:**

Healthy rats received two injections (at baseline and Week 4) of Dex-PLGA-Ms into the anterior chamber of the right eye. Clinical signs and intraocular pressure (IOP) were weekly recorded. The structure of the retina and optic nerve was *in vivo* evaluated using optical coherence tomography (OCT) every two weeks and functionally using dark- and light-adapted electroretinography at 0–12–24 weeks. Histological studies were also performed.

**Results:**

IOP progressively increased up to hypertension (23.22 ± 3.63 mmHg) in both eyes but did so later in left eyes. OCT quantified a decrease in full-thickness retina posterior pole (R), retinal-nerve-fiber layer (RNFL), and ganglion-cell layer (GCL) thickness up to 24 weeks. Right eyes showed higher neuroretinal thickness loss up to week 8. RNFL experienced the highest percentage thickness loss at the inferior-superior axis, while in GCL the inner sectors of the horizontal axis (Nasal-Temporal) suffered the greatest decrease in thickness. Retinal ganglion cell, photoreceptor, and intermediate cell functionality decreased over time. Increased deposition of collagen IV was also found in zonular fibers and the ciliary body.

**Conclusions:**

This work shows the usefulness of drug delivery systems, not to treat pathology but to induce it. Only two injections of Dex-PLGA-Ms in the anterior chamber of rat eyes were enough to progressively create ocular hypertension and subsequent functional and structural neuroretinal degeneration, at least over 6 months.

## Introduction

Ocular hypertension (OHT) has been associated with treatment with corticosteroids, such as dexamethasone, administered via topical eye drops or ointments (Chan and Salmon, 2019) or via subconjunctival, subtenon (Li et al., 2019) or intravitreous injections (Zarei-Ghanavati et al., 2012; Storey et al., 2018). When OHT is maintained over time, in some cases, this side effect produces what is known as steroid-induced glaucoma (SIG). Most patients decrease IOP and return to normal values in a few weeks after stopping the corticosteroid exposition, but sometimes patients develop a difficult-to-treat SIG (Kersey and Broadway, 2006) that in many cases requires eye surgery. The incidence of iatrogenic SIG has increased in recent years due to the increase in corticosteroid treatments for posterior pole pathologies with macular edema.

SIG shares characteristics with primary open-angle glaucoma (POAG). In fact, patients with POAG or susceptible to glaucoma (Fini et al., 2017), as well as their descendants, have a greater corticosteroid response, and a correlation has been demonstrated between the response to corticosteroids and the risk of developing glaucoma over time (Chan et al., 2019).

To study SIG, *ex vivo* models were developed (Torrejon et al., 2016; Rybkin et al., 2017). However, none of them fully simulate *in vivo* complexity. Several OHT models were therefore created in 8 non-human species (Overby and Clark, 2015), but only the rat (Razali et al., 2015) and mouse (Zode et al., 2014; Li et al., 2019) models developed glaucoma with functional and structural neuroretinal degeneration over a maximum follow-up of 2 months. These models have facilitated understanding of the histological, proteomic, and genetic characteristics of SIG, but many unknowns remain. It is known that corticosteroids increase the production of myocilin, fibronectin, or other proteins of the extracellular matrix (Faralli et al., 2015; Shan et al., 2017), and inhibit the metalloproteinase that leads to excessive accumulation of extracellular matrix and hence the clogging of the trabecular meshwork. Corticosteroids also decrease phagocytic activity (Zeng et al., 2019) (the function of trabecular meshwork cells), which leads to an increase in retained material and rigidity in the trabecular meshwork (Raghunathan et al., 2015), altering the motility/regulation of the aqueous humor drainage pathways (Overby and Clark, 2015; Xin et al., 2017; Patel et al., 2019, Patel et al., 2018). However, all these studies have only been conducted over short periods that prevent analysis of dynamic changes in the pathology over time. For these reasons, recent studies demand better characterization and improvement of the current SIG models, with the aim of simulating human chronic glaucoma (Agarwal and Agarwal, 2017; Biswas and Wan, 2019; Pang and Clark, 2020).

In a recently published paper, a novel pre-trabecular model of chronic glaucoma was achieved by repeated injection of biodegradable poly lactic-co-glycolic acid (PLGA) microspheres into the ocular anterior chamber of healthy rats. The presence of blank microspheres across the iridocorneal angle and the trabecular meshwork produced a mechanical blockage able to induce sustained OHT over six months. This continuous elevation of intraocular pressure (IOP) translated into progressive degradation of the retina in a pattern similar to that found in human glaucoma, with the advantage, over other animal models, that the ocular surface remained healthy throughout the study, causing less animal pain and allowing *in vivo* retinal monitoring over the six months (Rodrigo et al., 2021).

Long-term corticosteroid-induced chronic glaucoma requires delivery of small amounts of the active substance close to the trabecular meshwork over prolonged periods. This can now be achieved thanks to the development of drug delivery systems.

This study presents a new animal model of chronic SIG induced using injections of biodegradable PLGA Ms loaded with dexamethasone in the anterior chamber of rat eyes. This method combines not only pharmacological blockage of aqueous humor exit induced by the constant presence of the corticosteroid in the proximity of the trabecular meshwork but also additional mechanical blockage of the trabecular meshwork produced by the presence of PLGA Ms in the intracameral space. To our knowledge, this is the first SIG model that permits short- and long-term study and simulates real clinical conditions in which chronic treatment with corticosteroid is needed, such as corneal transplant or pathologies of the posterior pole with macular edema (Razeghinejad and Katz, 2012; Kornmann and Gedde, 2016).

## Materials

Dexamethasone (Dex) was supplied by Sigma-Aldrich (St. Louis Mo., USA) at the highest purity available (>98%). Poly (D,L-lactide-co-glycolide) (PLGA) 50:50 (inherent viscosity: 0.16–0.24 dl/g) was obtained from Evonik España (Granollers, Spain). The other materials employed in microsphere (Ms) manufacture were polyvinyl alcohol 67,000 g/mol (PVA) (purchased from Merck KGaA (Darmstadt, Germany) and methylene chloride (obtained from PanReac AppliChem, Barcelona, Spain).

## Methods

### Manufacture of dexamethasone-loaded PLGA microspheres

Dexamethasone-loaded PLGA Ms were obtained by applying the solvent extraction-evaporation method to an oil-in-water (O/W) emulsion. First, PLGA (400 mg) was dissolved in methylene chloride (2 mL). Micronized dexamethasone (40 mg) was added to the polymeric solution and dispersed by ultrasonication (Ultrasons; J.P. Selecta, Barcelona, Spain) for 5 minutes, then further sonicated (Sonicator XL; Heat Systems, Inc., Farmingdale, NY, USA) for 1 minute at 4 °C to obtain homogenous dispersion. The so-formed O-phase was emulsified by adding 5 mL of PVA MilliQ^®^ water solution 1% (w/v) via a homogenizer (Polytron^®^RECO, Kinematica, GmbHT PT3000, Lucerna, Switzerland) at 7,000 rpm for 1 minute. The resulting O/W emulsion was added to 100 mL of PVA MilliQ^®^ water solution 0.1% (w/v) and stirred for 3 hours leading to Ms maturation.

**Figure 1.**
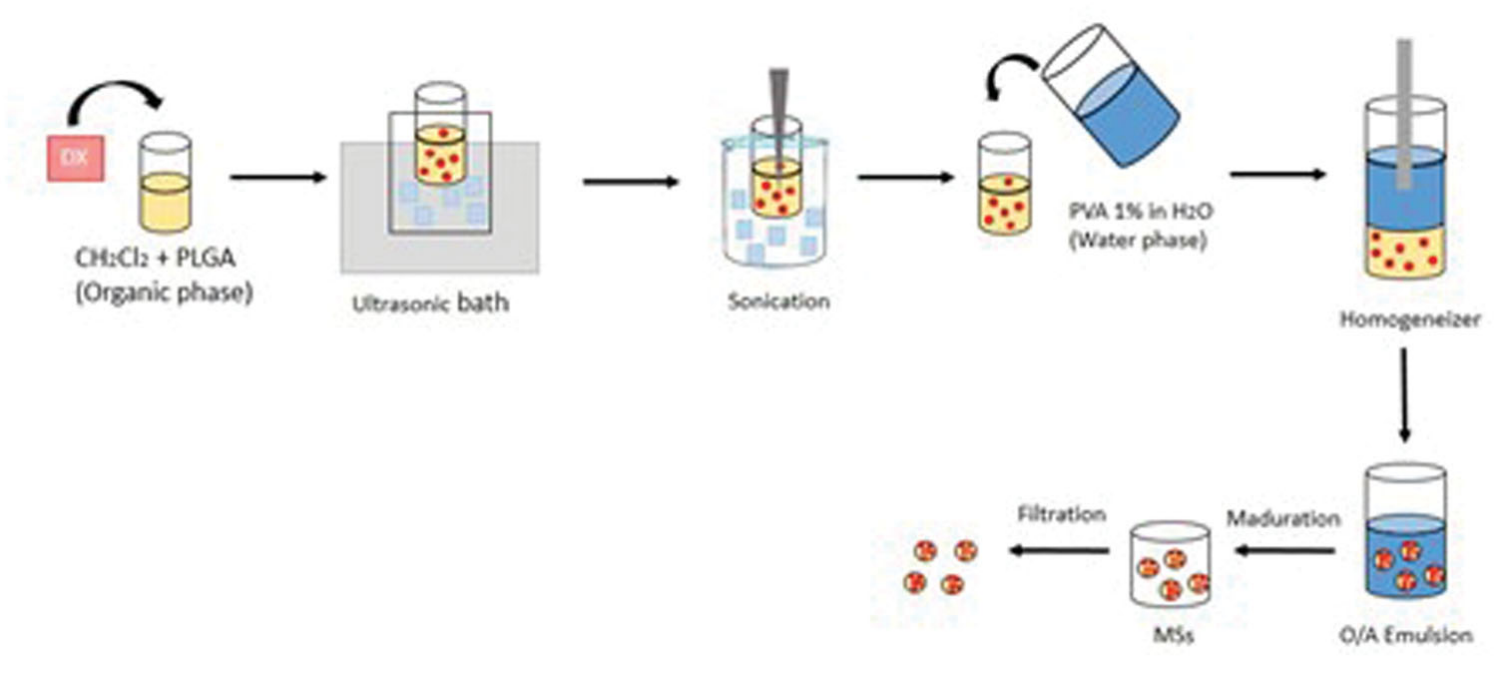
Dexamethasone-loaded PLGA microsphere manufacture.

Once maturation was completed, MilliQ^®^ water was employed to wash the Ms, removing the remaining superficial PVA. Subsequently, the 20–10 µm fraction was selected, freeze-dried (freezing: −60 °C/15 min.; drying: −60 °C/12 h/0.1 mBar) and stored at −30 °C in dry conditions (Figure 1).

### Dexamethasone-loaded PLGA microsphere characterization

Ms characterization was performed in terms of morphological evaluation, mean particle size, particle size distribution, encapsulation efficiency, and *in vitro* release. The production yield percentage of the selected granulometric fraction was also determined.

### Production yield percentage (PY %)

The production yield percentage was calculated according to the following Equation (1).

(1) $$\text{PY \%}=\frac{\text{amount of microspheres }}{\text{Total amount of polymer}+\text{Total amount of drug }}\text{ × }100$$

### External morphological evaluation

Gold sputter-coated freeze-dried dexamethasone-loaded PLGA Ms were observed using scanning electron microscopy (SEM; Jeol, JSM-6335F, Tokyo, Japan).

### Mean particle size and particle size distribution

Particle size and particle size distribution were measured using dual light scattering (Microtrac® S3500 Series Particle Size Analyzer, Montgomeryville, PA, USA). The volume means diameters ( ± standard deviation) obtained from 3 measurements were used to express the mean particle size.

### Dexamethasone quantification by HPLC/Ms

The HPLC/Ms system comprised a liquid chromatography instrument (Waters 1525 binary HPLC pump and Waters 2707 autosampler) employing a Nova-Pak C18 column (4 μm, ID 2.1 mm × 150 mm) coupled to a guard column (4 μm, 3.9 mm × 20 mm), both maintained at 45 °C. A Ms detector (Waters 3100 single quadrupole mass spectrometer) was connected to this system via an Empower 2 (Waters, Milford, USA). The ESI source was adjusted in the positive ion mode (ESI(+)) for DX detection. Selected ion recordings (SIR) dexamethasone mass (m/z) 393.40 was measured under mass spectrometer source conditions of 3.5 kV electrospray voltage and 130 °C heated capillary temperature. Nebulization (100 L/h flow rate, 130 °C source temperature, 5 V extractor voltage) and desolvation (400 L/h flow rate, 300 °C desolvation temperature) were performed employing nitrogen gas (>99.999%). An isocratic HPLC method was developed to quantify dexamethasone encapsulation efficiency and release from the Ms. This method comprised 50% ammonium acetate 15 mM/1 mL formic acid in MilliQ^®^ water and 50% acetonitrile at a flow rate of 0.3 mL/min.

### Dexamethasone encapsulation efficiency

A known quantity of dexamethasone-loaded PLGA Ms (1 mg) was dissolved in 2.5 mL of methylene chloride. Then, 6 mL of methanol were added to precipitate the dissolved polymer. After vortex mixing and centrifugation (5,000 rpm for 5 minutes at 20 °C), the upper methanolic supernatant was collected, filtered (0.22 µm), and analyzed using the previously described HPLC/Ms method in order to quantify the dexamethasone.

### *In vitro* study of dexamethasone release from dexamethasone-loaded PLGA Ms

A dexamethasone-loaded PLGA Ms suspension (2.5 mg/mL) was prepared in quadruplicate using phosphate-buffered saline (PBS, pH 7.4) with sodium azide (0.02% (w/v) as the release media. These samples were placed in a water shaker bath (100 rpm, 37 °C, Memmert Shaking Bath, Memmert, Schwabach, Germany). Supernatants were periodically collected after gentle centrifugation (5,000 rpm for 5 min, 20 °C) and filtered (0.22 μm) for dexamethasone quantification by HPLC/Ms employing the method previously mentioned. The remaining Ms samples were refilled with fresh release media. The same protocol was performed at each time point.

In order to mimic the animal study, the second dose of 2.5 mg of dexamethasone-loaded PLGA Ms was added to each sample at 28 days post-study (time of the second injection), also increasing the amount of release media to maintain the initial suspension concentration. The release study was performed as explained above for 140 additional days (total time of *in vitro* release study: 168 days, i.e. 24 weeks).

### Animals

The work with animals was approved by the Ethics Committee for Animal Research (PI34/17) and was carried out at the Aragón Biomedical Research Center (CIBA) in strict accordance with the ARVO Statement on the Use of Animals in Ophthalmic and Vision Research. The animals were housed in a light/dark-cycled room (12 hours light/12 hours dark) under controlled temperature (22 °C) and relative humidity (55%) conditions and in standard cages with environmental enrichment, water, and food *ad libitum*. Forty-three Long–Evans rats (40% males, 60% females; 4 weeks old; initial weights ranging from 50 to 100 g) were used for this longitudinal and interventionist study.

### Ocular hypertension (OHT) induction

At baseline and Week 4, all animals received a right-eye (RE) ocular hypertensive injection administered by superotemporal corneal puncture using a micro-metered Hamilton® syringe with a glass micropipette to insert 2 microlitres of dexamethasone-PLGA-Ms suspension into the anterior chamber of the eye.

This was performed under the following conditions: a sedative gas mixture containing 3% sevoflurane gas and 1.5% oxygen, topical eye drops containing tetracaine (1 mg/ml) + oxybuprocaine (4 mg/ml) (Anestesico Doble Colircusi®, Alcon Cusí® SA, Barcelona, Spain), analgesic subcutaneous dilution 1/10 of buprenorphine (0.05 mg/kg), aseptic surgical conditions achieved with the iodized solution and antibiotic eye drops containing ofloxacin (3 mg/ml) (Exocin Colircusi®, Alcon Cusí® SA, Barcelona, Spain). The temperature was controlled with warm pads and after the procedures, the animals were left to recover in a 2.5% oxygen-enriched atmosphere. The ophthalmological examination methodology was as described in (Rodrigo et al., 2021) as follows:

### Ophthalmological studies

#### In vivo

Clinical signs such as ocular surface redness, corneal alterations, intraocular inflammation, infection or cataract formation, as well as IOP measurements using the Tonolab® tonometer (Tonolab; Tiolat Oy Helsinki, Finland), were recorded by the same operator every week. A sedative mixture of 3% sevoflurane gas and 1.5% oxygen was applied, as recommended, for less than 3 minutes in order to avoid hypotensive effects (Ding et al., 2011). The IOP value was the average of three consecutive measurements, which resulted from the average of 6 rebounds.

#### Electroretinography

Neuroretinal structure functionality was studied using electroretinography (ERG) (Roland consult® RETIanimal ERG, Germany), applying the flash scotopic ERG and light-adapted (LA) photopic negative response (PhNR) protocols at baseline, Week 12 and Week 24. To perform the scotopic ERG test, the animals were dark-adapted for 12 hours and their pupils were fully dilated with tropicamide (10 mg/ml) and phenylephrine (100 mg/ml), (Alcon Cusí® SA, Barcelona, Spain) topically anesthetized with eye drops containing tetracaine (1 mg/ml) + oxybuprocaine (4 mg/ml) (Anestesico Doble Colircusi®, Alcon Cusí® SA, Barcelona, Spain) and lubricated with hypromellose 2% (Methocel® OmniVision, Germany). Active electrodes were placed on the cornea, references were placed on either side under the skin and the ground electrode was placed near the tail. Electrode impedance with a difference of <2 kΩ between electrodes was accepted. Both eyes were simultaneously tested using a Ganzfeld Q450 SC sphere with white LED flashes for stimuli, and seven steps at increasing luminance intensity and intervals were performed. Scotopic test-examined rod response: step 1: −40 dB, 0.0003 cds/m^2^, 0.2 Hz [20 recordings averaged]; step 2: −30 dB, 0.003 cds/m^2^, 0.125 Hz [18 recordings averaged]; step 3: −20 dB, 0.03 cds/m^2^, 8.929 Hz [14 recordings averaged]; step 4: −20 dB, 0.03 cds/m^2^, 0.111 Hz [15 recordings averaged]; step 5: −10 dB, 0.3 cds/m^2^, 0.077 Hz [15 recordings averaged]; mixed rod–cone response: step 6: 0 dB, 3.0 cds/m^2^, 0.067 Hz [12 recordings averaged]; and oscillatory potentials: step 7: 0 dB, 3.0 cds/m^2^, 29.412 Hz [10 recordings averaged]). The PhNR protocol was performed after light adaptation to a blue background (470 nm, 25 cds/m^2^), and a red LED flash (625 nm, −10 dB, 0.30 cds/m^2^, 1.199 Hz [20 recordings averaged]) was used as stimulus. Latency (in milliseconds) and amplitude (in microvolts) were studied in a, b and PhNR waves.

#### Optical coherence tomography

Neuroretinal structures were analyzed using optical coherence tomography (OCT Spectralis, Heidelberg® Engineering, Germany) at 0, 2, 4, 6, 8, 12, 18, and 24 weeks applying a contact lens adapted to the rat cornea to acquire higher quality images. The full-thickness Retina posterior pole (R), Retina Nerve Fiber Layer (RNFL), and Ganglion Cell Layer (GCL) protocols were used with automatic segmentation. These protocols analyzed a circled area, centered on the optic disk, by means of 61 b-scans. Subsequent follow-up examinations were performed at this same location using the eye-tracking software and follow-up application. The Retina and GCL were analyzed via the 9 ETDRS areas, which included a central (C) 1 mm circle centered on the optic disk (though no fovea exists in rats) and inner (inferior -II-, superior -IS-, nasal -IN-, temporal -IT-) and outer (inferior -OI-, superior -OS, nasal -ON-, temporal -OT-) rings measuring 2 and 3 mm in diameter, respectively. Total volume (TV) was also measured. Retinal thickness comprises from the inner limiting membrane to the retinal pigment epithelium, and the GCL comprises from the RNFL to the inner nuclear layer boundaries. The RNFL protocol provided measurements of the 6 peripapillary sectors (inferotemporal -IT-, inferonasal -IN-, superotemporal -ST-, superonasal -SN-, nasal -N- and temporal -T-), measuring the thickness from the inner limiting membrane to the GCL boundaries.

Each OCT scan was revised by the same researcher. The image was manually corrected if detected biased examinations due to errors in segmentation, excessive noise, or artifact. It occurred very occasionally. To perform the ERG and OCT tests, the animals were intraperitoneally anesthetized with a mixture of ketamine (60 mg/kg) + dexmedetomidine (0.25 mg/kg) and temperature controlled. At the end of the study, six unconscious animals were euthanized under humane conditions with an intracardiac injection of sodium thiopental (25 mg/ml) and their eyes were immediately enucleated.

### Histology

Paraffin-embedded eyes were sectioned (5 µm) along the eye axis, deparaffinized, and rehydrated. After several washes in PBS, the sections were incubated overnight at 4 °C with the following primary antibodies: mouse anti-Brn3a (Santa Cruz Biotechnology, Heidelberg, Germany) at 1:50 dilution; goat anti-mouse Collagen IV (Milllipore, Temecula, USA) at 1:20 dilution; and rabbit anti-mouse laminin (DAKO, Glostrup, Denmark) at 1:200 dilution. After washing the sections in PBS, the slides were incubated for 2 hours at room temperature with the secondary rabbit anti-goat Alexa 488 and anti-rabbit Alexa 488 antibodies (Invitrogen, Carlsbad, CA, USA) at 1:100 dilution. Propidium iodide (Sigma-Aldrich Corp., St. Louis, MO, USA) diluted in PBS (1  :  10) was used for nuclear counterstaining. The slides were mounted in Fluoromount (Sigma-Aldrich) medium for further analysis using a laser scanning confocal microscope (TCS SP5; Leica Microsystems GmbH, Heidelberg, Germany). Immunohistochemistry controls were performed by omission of the primary antibody in a sequential tissue section.

For ganglion cell count, slides probed with mouse anti-Brn3a were incubated with biotinylated horse anti-mouse at 1:50 dilution (Vector Laboratories, Burlingame, CA, USA). They were then incubated with ABC-HRP (Thermo Fisher Scientific, Waltham Massachusetts, USA) at 1:50 dilution at room temperature. The sections were washed in PBS before and after every incubation. Finally, the sections were stained with DAB (Sigma-Aldrich) for 3 minutes and counterstained with Harris’ hematoxylin (Sigma-Aldrich) for 20 minutes at room temperature. Ganglion cells were counted in radial sections of the retina along 2 mm of a linear region of the ganglion cell layer corresponding to four areas, two on each side of the optic nerve head. The images were analyzed by an operator blinded to treatment groups.

### Statistical analysis

The data were recorded in an Excel database and statistical analysis was performed using SPSS software version 20.0 (SPSS Inc., Chicago, IL). The Kolmogorov-Smirnov test was used to assess sample distribution, Student’s *t*-test was used to evaluate the differences between eyes, and a paired Student’s *t*-test was used to compare the changes recorded in each eye over the study period. All values were expressed as mean ± standard deviations. Values of *p* < .05 (expressed as *) were considered to indicate statistical significance. The Bonferroni correction for multiple comparisons was also calculated to avoid a high false-positive rate. The level of significance for each variable was established according to Bonferroni calculations (expressed as ^#^).

## Results

### Dexamethasone-loaded PLGA microsphere characterization

**Figure 2.**
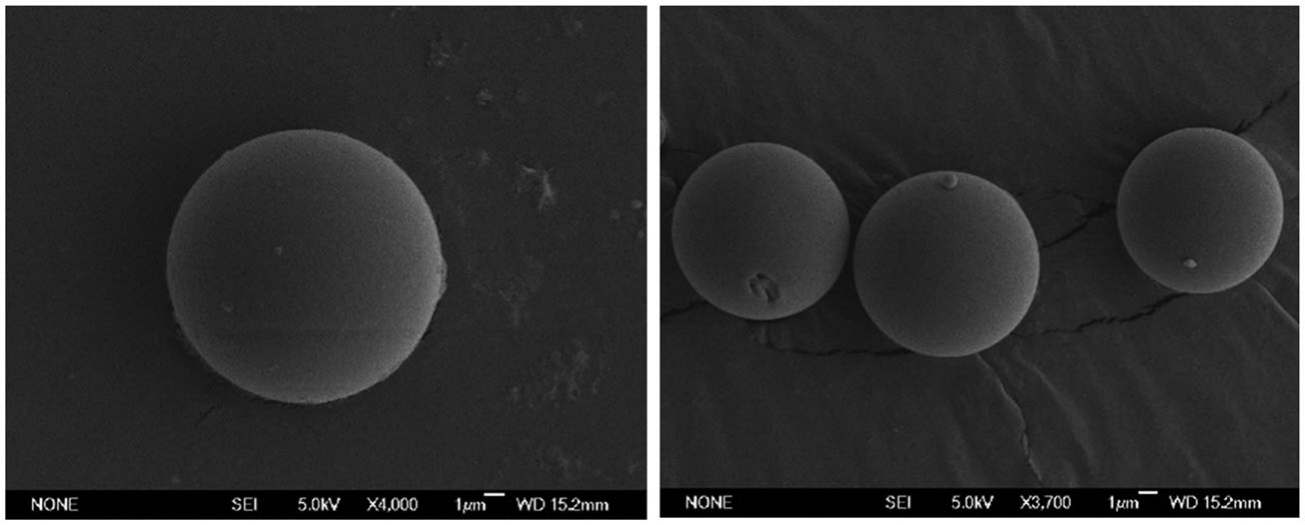
SEM pictures of dexamethasone-loaded PLGA Ms.

Dexamethasone-loaded PLGA Ms showed a production yield of 77.34% for the 20–10 µm fraction. The particle size distribution was unimodal, with a mean particle size of 13.13 ± 0.60 µm. Drug loading was 60.70 ± 1.03 µg dexamethasone/mg Ms, constituting 66.77 ± 1.14% encapsulation efficiency. SEM images (Figure 2) evidenced the presence of spherical non-porous Ms surfaces.

### *In vitro* release studies

**Figure 3.**
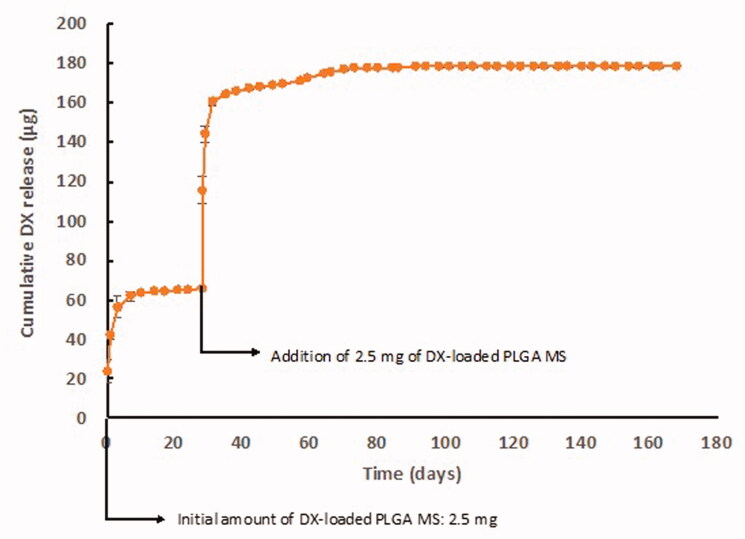
*In vitro* dexamethasone release profile from dexamethasone-loaded PLGA Ms.

The *in vitro* release profile of dexamethasone from PLGA microspheres showed the typical multiphasic shape combining rapid- and slow-release periods. From Day 0 to Day 7, the rapid release occurred leading to a total release of 62 µg. This first phase was followed by slow release up to Day 28, with an average release rate of 0.191 µg/day. After the inclusion of the additional amount of Ms in the release media, a second rapid-release occurred, delivering 94.5 µg in the following 3 days. Subsequently, a second slow-release rate of 0.390 µg/day was observed until Day 77 of the *in vitro* release study. No dexamethasone release was observed from Day 77 to study end at Day 168, but the release assay was continued to cover the complete six-month study (Figure 3).

### Ophthalmological clinical signs and intraocular pressure

Ocular injections were generally well tolerated. The visual axis remained clear and the iridocorneal angle was open, appearing normal when examined by light microscopy. As for disadvantages, four animals developed mild peripheral corneal leucomas that nevertheless did not preclude proper testing and follow-up, but one rat developed cataracts with pupillary seclusion and ocular hypotension and this animal was discarded from the results.

**Figure 4.**
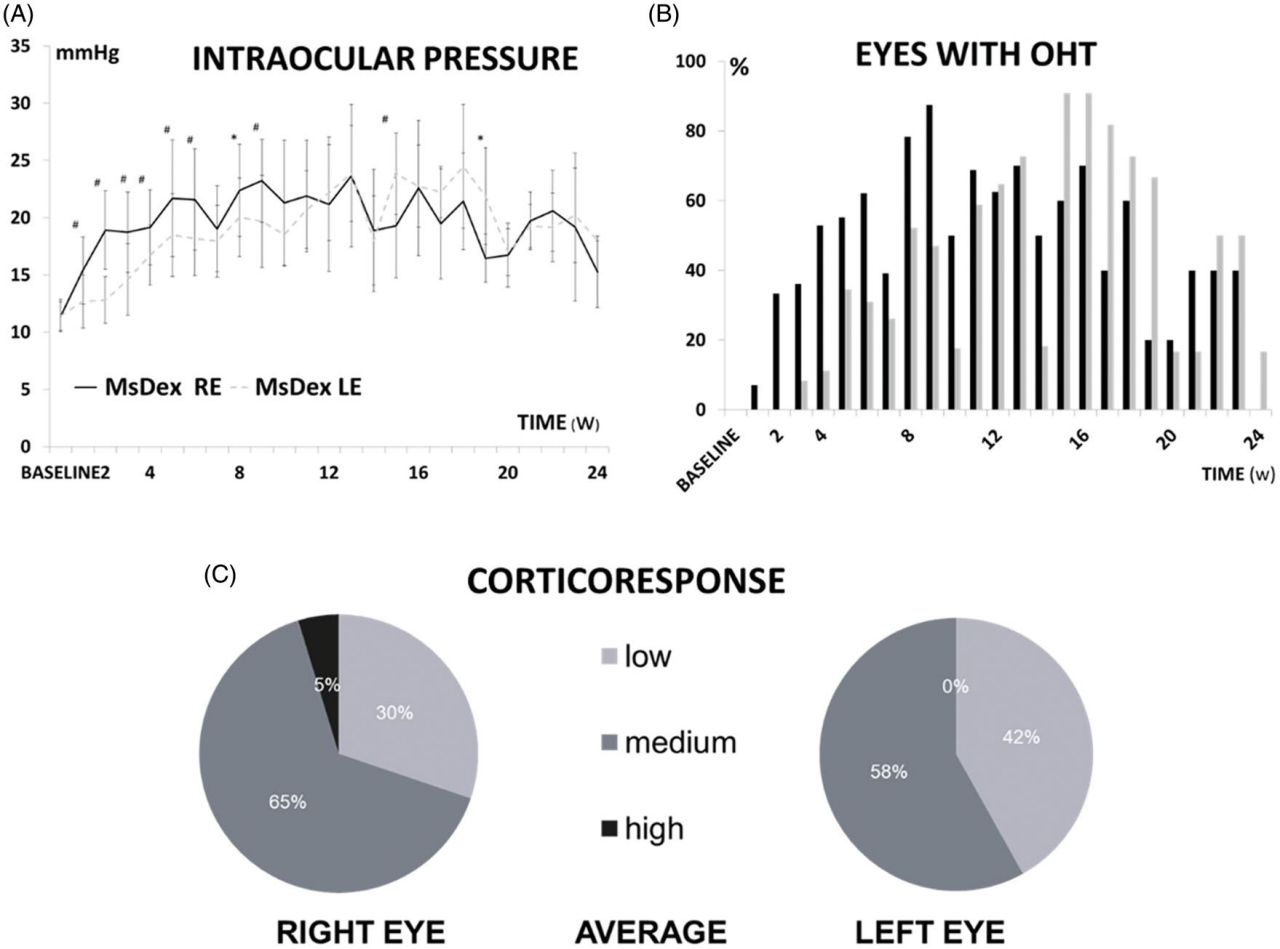
(a) Intraocular pressure curve over 6 months in the MsDex (dexamethasone-loaded microsphere) model. IOP: intraocular pressure; MsDex: microspheres loaded with dexamethasone; RE: right eye; LE: left eye; w: week; *p < 0.05; #*p* < 0.02. (b) Percentage of ocular hypertensive eyes (>20 mmHg) in MsDex model over 6 months of follow-up. OHT: ocular hypertension; %: percentage; MsDex: microspheres loaded with dexamethasone; RE: right eye; LE: left eye; w: week. (c) Percentage of corticosteroid response in right and left eyes. Low: <6 mmHg increase; medium: 6–15 mmHg increase; high: >15 mmHg increase.

At baseline, no differences in IOP were found between eyes and the IOP progressively increased over follow-up. Injected REs experienced an increase (4 mmHg; 36.30% increase) from Week 1 onwards, reached OHT (considered as >20 mmHg) at Week 5 (95.39% increase) and remained significantly higher than the contralateral left eye (LE) up to Week 9 (23.22 ± 3.63 vs 19.68 ± 4.03 mmHg, *p* = .013) (102.42% increase). LEs reached OHT at Week 8 (79.25% increase) and even surpassed it at later stages (16.46 ± 2.11 vs 21.88 ± 4.21 mmHg, *p* = .029) (Figure 4(a)). This model showed a sustained and increasing percentage of OHT in both eyes, albeit occurring 3 weeks later in LEs. REs reached the OHT percentage peak (87.5%) at Week 9 and an even higher level (90%) was quantified in LEs at 15–16 weeks (Figure 4(b)). Most rats experienced an IOP increase of between 6 and 15 mmHg (medium corticosteroid response) and only 5% on average showed an increase higher than 15 mmHg (high corticosteroid response) (Figure 4(c)).

### *In vivo* neuroretinal examination

#### Optical coherence tomography:

**Figure 5.**
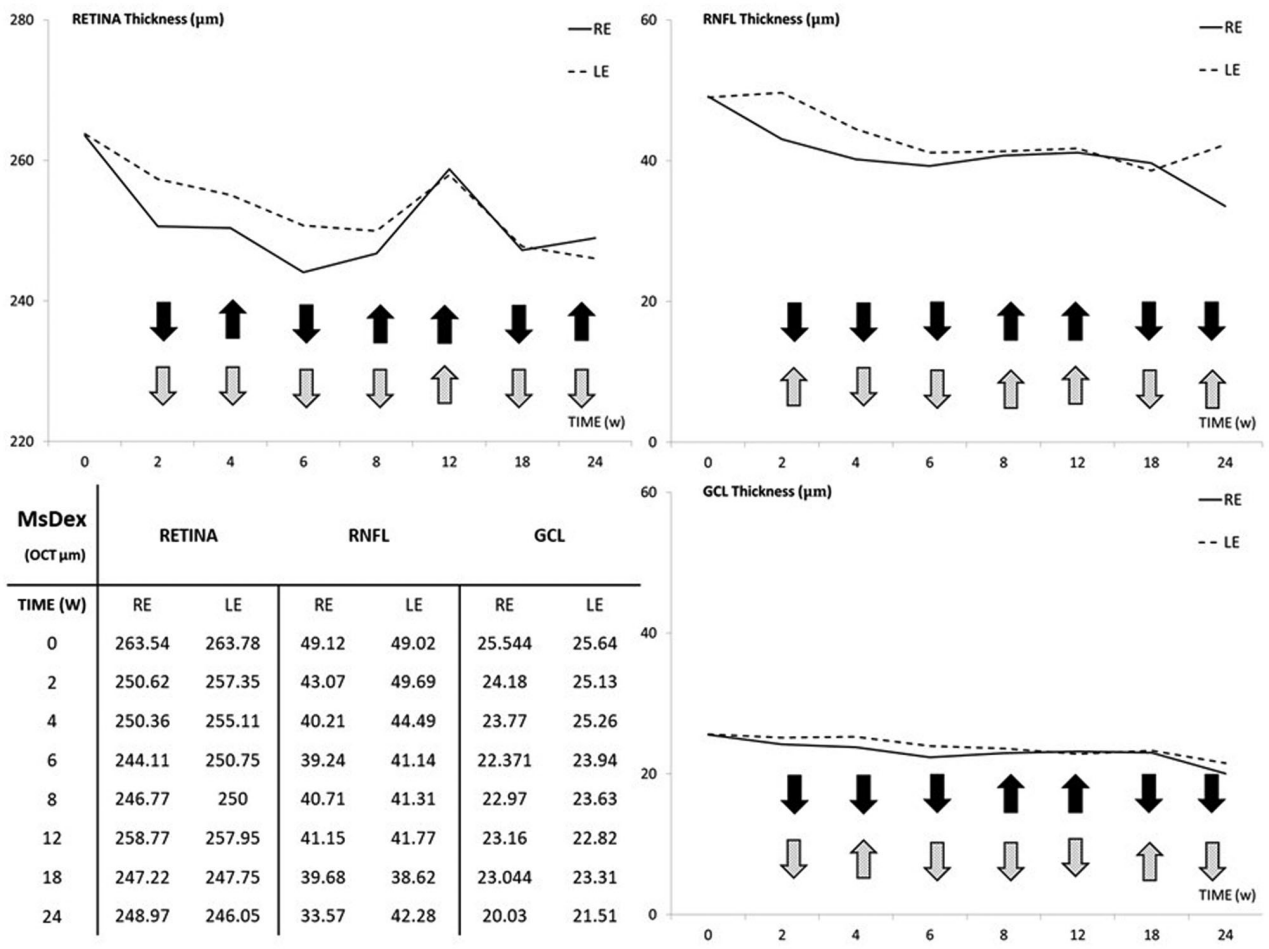
Structural analysis of the neuroretina, measured by optical coherence tomography, of both eyes in the dexamethasone-loaded microsphere (MsDex) model. MsDex: microspheres loaded with dexamethasone; RE: right eye; LE: left eye; w: week; OCT: optical coherence tomography; RNFL: Retina Nerve Fiber Layer; GCL: Ganglion Cell Layer; average thickness in microns (μm). Black arrows: from the right eye; gray arrows: from the left eye; down arrow: decreasing thickness; up arrow: increasing thickness.

Both eyes experienced a progressive decrease in thickness (in microns) in the retina, RNFL, and GCL over 6 months of follow-up. REs showed lower thicknesses than LEs in all sectors explored (except nasal sectors), with statistical differences up to Week 8, although this tendency inverted at Weeks 12 and 18. By the end of the study period (Week 24), REs showed smaller thicknesses in all sectors in the RNFL and GCL but higher thicknesses in the Retina (Table 1). Fluctuations were observed in both eyes, with these being more evident in the retina and at Week 12. REs experienced the same fluctuation tendency in the RNFL and GCL, which occurred 2 weeks later in LEs (Figure 5).

**Table 1.** Structural neuroretinal analysis, measured by optical coherence tomography (OCT), using microspheres loaded with dexamethasone (MsDex) over 6 months.

OCT parameters	Baseline		2 w			4 w			6 w			8 w			12 w			18 w			24 w		
	Mean ± SD	*p*	Mean ± SD	%Ch	*p*	Mean ± SD	%Ch	*p*	Mean ± SD	%Ch	*p*	Mean ± SD	%Ch	*p*	Mean ± SD	%Ch	*p*	Mean ± SD	%Ch	*P*	Mean ± SD	%Ch	*p*
Retinal thickness																							
Central	288.90 ± 16.32	0.373	267.00 ± 26.90	−7.58	0.431	275.33 ± 18.49	−4.69	0.580	255.33 ± 19.18	−11.61	0.058	260.40 ± 19.61	−9.87	0.190	292.50 ± 17.74	1.25	0.461	262.00 ± 14.56	−9.31	0.780	260.60 ± 24.05	−9.80	0.774
	295.20 ± 14.45		276.50 ± 9.02	−6.33		282.50 ± 24.45	−4.30		276.00 ± 13.82	−6.50		276.50 ± 18.06	−6.76		285.60 ± 9.99	−3.25		259.80 ± 8.75	−11.99		263.83 ± 11.08	−10.63	
Inner inferior	267.90 ± 8.71	0.668	257.33 ± 17.31	−3.94	0.254	262.33 ± 6.59	−2.07	0.443	250.33 ± 6.25	−6.55	0.316	255.60 ± 4.61	−4.59	0.575	259.83 ± 7.62	−3.01	0.709	255.20 ± 13.18	−4.74	0.804	249.80 ± 17.65	−6.76	0.980
	266.30 ± 7.64		267.00 ± 9.12	0.26		259.67 ± 4.84	−2.48		254.17 ± 6.33	−4.55		253.83 ± 5.30	−4.91		262.20 ± 12.59	−1.54		258.60 ± 26.50	−2.89		250.00 ± 6.63	−6.12	
Outer inferior	254.80 ± 6.59	0.955	244.50 ± 6.71	−4.04	0.269	244.50 ± 2.07	−4.04	0.561	240.17 ± 2.63	−5.74	0.254	240.20 ± 6.30	−5.73	0.856	244.00 ± 5.96	−4.24	0.543	239.20 ± 6.26	−6.12	0.875	241.40 ± 20.37	−5.26	0.596
	254.60 ± 8.82		248.50 ± 5.01	−2.39		245.67 ± 4.27	−3.50		242.33 ± 3.50	−4.81		241.00 ± 7.64	−5.64		247.40 ± 11.52	−2.83		238.00 ± 15.28	−6.52		236.50 ± 7.63	−7.11	
Inner superior	261.20 ± 7.81	0.897	245.50 ± 10.69	−6.01	0.209	242.00 ± 6.00	−7.35	0.039	238.83 ± 4.53	−8.56	0.005^#^	240.80 ± 8.46	−7.81	0.278	260.50 ± 14.30	−0.27	0.588	243.40 ± 7.19	−6.81	0.932	241.20 ± 12.55	−7.66	0.812
	261.60 ± 5.56		255.50 ± 14.78	−2.33		254.00 ± 10.84	−2.90		248.33 ± 4.63	−5.07		246.67 ± 8.33	−6.05		256.00 ± 11.76	−2.14		243.80 ± 7.19	−6.80		243.17 ± 13.84	−7.05	
Outer superior	260.00 ± 7.24	0.941	247.83 ± 8.03	−4.68	0.311	242.50 ± 2.73	−6.73	0.072	243.17 ± 4.66	−6.47	0.098	246.60 ± 5.94	−5.15	0.696	258.17 ± 7.78	−0.70	0.634	242.20 ± 7.53	−6.85	0.940	252.20 ± 12.35	−3.00	0.249
	259.70 ± 10.39		252.83 ± 8.18	−2.64		248.83 ± 7.22	−4.18		247.33 ± 3.07	−4.76		248.33 ± 7.89	−4.58		256.00 ± 6.55	−1.42		242.60 ± 8.79	−6.58		243.67 ± 10.63	−6.17	
Inner nasal	263.10 ± 7.63	0.810	250.00 ± 9.57	−4.97	0.139	250.33 ± 6.37	−4.85	0.010^#^	244.17 ± 6.52	−7.19	0.035	247.40 ± 7.47	−5.97	0.751	257.67 ± 10.13	−2.06	0.646	247.00 ± 7.96	−6.12	0.192	251.80 ± 7.49	−4.29	0.748
	264.00 ± 8.81		258.67 ± 9.11	−2.01		261.50 ± 5.82	−0.94		253.50 ± 6.74	−39.727		249.17 ± 9.90	−5.95		261.80 ± 18.29	−0.83		254.80 ± 9.28	−3.48		250.67 ± 3.55	−5.05	
Outer nasal	258.70 ± 7.11	0.659	246.83 ± 4.62	−4.58	0.715	244.33 ± 2.87	−5.55	0.536	239.33 ± 7.17	−7.48	0.294	244.00 ± 5.24	−5.68	0.882	250.17 ± 6.43	−3.30	0.938	241.20 ± 8.22	−6.76	0.973	248.75 ± 2.63	−3.85	0.083
	257.20 ± 7.81		248.00 ± 6.03	−3.57		245.83 ± 4.95	−4.42		242.83 ± 2.92	−5.58		244.50 ± 5.54	−5.19		250.60 ± 11.45	−2.57		241.40 ± 9.94	−6.14		241.00 ± 7.40	−6.30	
Inner temporal	261.60 ± 7.80	0.432	252.83 ± 9.90	−3.35	0.444	248.50 ± 8.01	−5.00	0.807	244.17 ± 5.19	−6.66	0.723	240.60 ± 6.84	−8.03	0.466	255.50 ± 11.45	−2.33	0.409	252.20 ± 11.32	−3.59	0.455	246.40 ± 11.03	−5.81	0.681
	258.80 ± 7.77		257.17 ± 8.88	−62		249.67 ± 8.06	−3.52		245.00 ± 2.09	−5.33		243.83 ± 7.13	−6.14		250.40 ± 7.02	−3.25		247.00 ± 9.56	−4.56		243.83 ± 9.04	−5.78	
Outer temporal	255.70 ± 7.36	0.761	243.83 ± 6.55	−4.64	0.046	243.50 ± 3.27	−4.77	0.021	241.50 ± 5.82	−5.55	0.052	245.40 ± 8.41	−4.03	0.874	250.67 ± 8.71	−1.97	0.830	242.60 ± 9.18	−5.12	0.836	248.60 ± 6.50	−2.78	0.135
	256.70 ± 7.10		252.00 ± 5.86	−1.83		248.33 ± 2.80	−3.26		247.33 ± 2.87	−3.65		246.17 ± 7.19	−4.28		251.60 ± 3.84	−1.99		243.80 ± 8.52	−5.03		241.83 ± 7.02	−5.79	
Total volume	1.85 ± 0.04	0.852	1.76 ± 0.06	−4.90	0.127	1.76 ± 0.35	−4.90	0.194	1.72 ± 0.02	−7.23	0.017^#^	1.74 ± 0.05	−6.03	0.549	1.82 ± 0.05	−1.67	0.892	1.74 ± 0.05	−6.14	0.999	1.71 ± 0.13	−7.76	0.696
	1.86 ± 0.04		1.81 ± 0.03	−2.41		1.79 ± 0.43	−3.40		1.76 ± 0.02	−5.10		1.76 ± 0.05	−5.38		1.82 ± 0.06	−2.15		1.74 ± 0.06	−6.34		1.73 ± 0.04	−6.72	
RNFL thickness																							
GLOBAL	49.10 ± 5.34	0.963	43.00 ± 3.52	−12.42	0.059	40.17 ± 3.06	−18.18	0.122	39.50 ± 3.20	−19.55	0.411	40.40 ± 1.14	−17.72	0.368	40.33 ± 3.67	−17.86	0.640	39.60 ± 4.33	−19.35	0.575	33.40 ± 8.47	−31.98	0.097
	49.00 ± 3.94		49.67 ± 6.80	1.36		44.33 ± 5.20	−9.53		41.00 ± 2.82	−16.32		41.17 ± 1.47	−19.02		41.60 ± 5.03	−15.10		38.40 ± 1.51	−21.63		41.83 ± 6.64	−14.63	
Inferior temporal	51.30 ± 6.53	0.758	44.50 ± 8.26	−13.25	0.678	35.17 ± 6.82	−31.44	0.042	32.67 ± 9.35	−36.31	0.200	44.40 ± 6.42	−13.45	0.214	42.67 ± 10.15	−16.82	0.912	45.60 ± 27.68	−11.11	0.559	37.80 ± 9.75	−26.32	0.094
	52.30 ± 7.68		46.50 ± 7.91	−11.08		42.83 ± 4.26	−18.10		38.17 ± 2.99	−27.01		39.83 ± 4.91	−31.31		43.40 ± 11.14	−17.02		38.00 ± 3.39	−27.34		51.33 ± 13.42	−1.85	
Inferior nasal	51.70 ± 4.83	0.513	43.67 ± 6.86	−15.53	0.073	41.67 ± 2.58	−19.40	0.026	42.67 ± 8.26	−17.46	0.664	45.80 ± 5.35	−11.41	0.568	50.50 ± 8.96	−2.32	0.633	36.40 ± 6.02	−29.59	0.506	43.00 ± 11.42	−16.83	0.476
	49.30 ± 10.29		55.33 ± 12.53	12.23		47.67 ± 5.00	−3.30		40.67 ± 7.20	−17.50		43.17 ± 8.61	−14.20		47.40 ± 11.88	−3.85		39.80 ± 9.09	−19.27		49.33 ± 15.87	0.06	
Superior temporal	51.90 ± 6.72	0.849	43.83 ± 12.28	−15.54	0.166	51.33 ± 11.74	−1.09	0.624	41.50 ± 11.52	−20.03	0.207	41.20 ± 3.76	−20.62	0.388	40.00 ± 13.19	−22.93	0.859	42.60 ± 6.46	−17.92	0.491	30.40 ± 18.91	−41.43	0.486
	52.60 ± 9.30		55.17 ± 13.93	4.88		48.33 ± 8.54	−8.11		49.17 ± 7.78	−6.52		46.00 ± 11.24	−14.35		41.40 ± 12.03	−21.29		45.20 ± 4.81	−14.07		38.83 ± 19.38	−26.18	
Superior nasal	40.30 ± 12.07	0.725	41.67 ± 8.95	3.39	0.732	34.00 ± 3.74	−15.63	0.075	37.67 ± 5.20	−6.52	0.949	32.80 ± 6.14	−18.61	0.401	33.60 ± 9.65	−16.63	0.765	36.20 ± 3.11	−10.17	0.270	25.40 ± 7.47	−36.97	0.052
	42.20 ± 11.67		43.17 ± 5.34	2.29		40.17 ± 6.61	−4.81		37.83 ± 3.43	−10.35		36.67 ± 8.01	−15.08		35.20 ± 6.38	−16.59		34.00 ± 2.73	−19.43		33.83 ± 4.99	−19.83	
Nasal	48.20 ± 10.62	0.142	40.83 ± 4.11	−15.29	0.359	40.50 ± 2.88	−15.97	0.948	39.50 ± 3.56	−18.04	0.156	41.40 ± 6.65	−14.11	0.611	40.67 ± 3.77	−15.62	0.398	37.00 ± 6.28	−23.24	0.475	32.20 ± 10.28	−33.20	0.194
	42.50 ± 5.01		43.67 ± 5.92	27.576		40.33 ± 5.35	−5.10		36.50 ± 3.20	−14.11		39.50 ± 5.32	−7.59		36.80 ± 9.93	−13.41		34.60 ± 3.43	−18.59		39.67 ± 7.36	−6.66	
TEMPORAL	51.40 ± 11.66	0.413	44.00 ± 3.74	−14.39	0.037	38.67 ± 5.61	−24.76	0.032	41.17 ± 10.96	−19.90	0.498	39.00 ± 2.55	−24.12	0.043	40.33 ± 4.32	−21.54	0.162	40.40 ± 4.77	−21.40	0.999	32.80 ± 11.03	−36.19	0.125
	55.30 ± 8.97		54.33 ± 9.85	−1.75		47.83 ± 7.05	−13.50		44.67 ± 5.31	−19.22		42.83 ± 2.78	−29.12		46.60 ± 8.96	−15.73		40.40 ± 3.13	−26.94		41.17 ± 4.75	−25.55	
GCL THICKNESS																							
CENTRAL	22.00 ± 2.70	0.827	20.67 ± 4.27	−6.04	0.999	18.67 ± 3.14	−15.13	0.139	18.17 ± 2.78	−17.40	0.225	18.00 ± 2.73	−18.18	0.415	21.00 ± 2.82	−4.55	0.067	18.60 ± 2.51	−15.45	0.819	17.40 ± 2.40	−20.91	0.291
	21.70 ± 3.30		20.67 ± 1.86	−4.74		21.00 ± 1.67	−3.22		20.33 ± 3.01	−6.31		19.50 ± 3.01	−11.28		17.60 ± 2.51	−18.89		19.00 ± 2.82	−12.44		18.83 ± 1.83	−13.23	
INNER INFERIOR	27.10 ± 2.13	0.671	26.67 ± 3.67	−1.58	0.497	26.83 ± 2.04	−0.99	0.726	24.83 ± 1.32	−8.37	0.172	25.40 ± 1.14	−6.27	0.737	25.50 ± 1.64	−5.90	0.749	25.60 ± 3.36	−5.54	0.738	22.80 ± 2.38	−15.87	0.022
	27.50 ± 2.01		27.83 ± 1.72	1.2		27.17 ± .98	−1.2		26.00 ± 1.41	−5.45		25.67 ± 1.36	−7.13		25.80 ± 1.30	−6.18		26.20 ± 1.92	−4.73		25.83 ± 1.16	−6.07	
OUTER INFERIOR	26.10 ± 1.79	0.819	25.33 ± 1.03	−2.95	0.260	25.00 ± 1.54	−4.21	0.172	23.50 ± 2.34	−9.96	0.091	23.40 ± 2.60	−10.34	0.967	24.50 ± 1.87	−6.13	0.531	23.00 ± 3.53	−11.88	0.324	21.40 ± 3.43	−18.01	0.135
	26.30 ± 2.05		26.00 ± 0.8	−1.14		26.17 ± 1.16	−0.49		25.33 ± 0.51	−3.68		23.33 ± 2.58	−12.73		25.20 ± 1.64	−4.18		24.80 ± 1.48	−5.70		23.83 ± 1.16	−9.39	
INNER SUPERIOR	25.70 ± 2.83	0.682	23.00 ± 2.75	−10.50	0.165	22.67 ± 2.80	−11.78	0.169	19.50 ± 2.16	−24.12	0.012	21.20 ± 2.38	−17.51	0.035	23.00 ± 1.78	−10.51	0.999	21.20 ± 4.65	−17.51	0.945	18.20 ± 0.83	−29.18	0.653
	26.10 ± 1.10		25.17 ± 2.22	−3.56		24.67 ± 1.75	−5.47		23.33 ± 2.16	−10.61		23.67 ± 0.51	−10.27		23.00 ± 3.74	−11.88		21.00 ± 4.30	−19.54		18.83 ± 2.92	−27.85	
OUTER SUPERIOR	24.70 ± 2.79	0.710	24.50 ± 2.074	−0.80	0.892	23.83 ± 1.47	−3.52	0.064	24.17 ± 2.71	−2.14	0.528	24.60 ± 2.19	−0.40	0.464	24.00 ± 3.79	−2.83	0.615	25.00 ± 2.55	1.21	0.636	19.40 ± 2.60	−21.46	0.104
	25.10 ± 1.85		24.67 ± 2.06	−1.711		25.83 ± 1.83	2.90		25.00 ± 1.54	−0.39		25.33 ± 0.81	0.91		22.60 ± 5.12	−9.96		24.20 ± 2.58	−3.59		22.33 ± 2.73	−11.04	
INNER Nasal	25.60 ± 2.31	0.916	23.00 ± 4.19	−10.15	0.215	23.50 ± 2.34	−8.20	0.260	21.50 ± 3.93	−16.01	0.401	22.80 ± 1.30	−10.94	0.728	21.67 ± 3.26	−15.35	0.498	22.80 ± 2.28	−10.94	0.551	19.00 ± 1.41	−25.78	0.293
	25.70 ± 1.82		25.33 ± 1.03	−1.43		25.33 ± 2.94	−1.43		23.33 ± 3.26	−9.22		23.17 ± 1.94	−10.92		23.00 ± 2.91	−10.51		21.80 ± 2.77	−15.18		20.17 ± 1.94	−21.52	
Outer Nasal	26.10 ± 1.66	0.558	25.17 ± 1.47	−3.56	0.169	25.50 ± 1.37	−2.29	0.679	23.17 ± 2.48	−11.22	0.178	24.40 ± 1.34	−6.51	0.596	22.50 ± 3.01	−13.79	0.559	23.00 ± 2.44	−11.88	0.550	19.75 ± 1.25	−24.33	0.337
	25.60 ± 2.06		24.17 ± 0.7	−5.58		25.17 ± 1.32	−1.67		24.83 ± 1.32	−3.00		23.83 ± 1.94	−7.43		23.60 ± 2.96	−7.81		23.80 ± 1.48	−7.03		21.00 ± 2.19	−17.97	
Inner temporal	26.30 ± 2.26	0.777	24.00 ± 2.96	−8.74	0.432	23.33 ± 1.86	−11.29	0.056	22.00 ± 2.28	−16.34	0.401	22.40 ± 2.60	−14.83	0.527	22.33 ± 0.81	−15.10	0.409	23.20 ± 2.77	−11.79	0.497	21.40 ± 2.30	−18.63	0.599
	26.00 ± 2.40		25.17 ± 1.83	−3.19		25.83 ± 2.13	−0.65		23.33 ± 2.94	−10.26		23.50 ± 2.88	−10.64		21.20 ± 3.11	−18.46		24.20 ± 1.48	−6.92		20.67 ± 2.16	−20.50	
Outer temporal	26.30 ± 2.21	0.610	25.33 ± 2.42	−3.68	0.144	24.67 ± 1.63	−6.19	0.112	24.50 ± 2.07	−6.84	0.746	24.60 ± 1.14	−6.46	0.956	24.00 ± 2.28	−8.75	0.808	25.00 ± 2.73	−4.94	0.897	21.00 ± 3.53	−20.15	0.455
	26.80 ± 2.09		27.17 ± 1.47	1.38		26.17 ± 1.32	−2.35		24.00 ± 3.03	−10.44		24.67 ± 2.42	−8.63		23.40 ± 5.36	−12.69		24.80 ± 1.92	−7.46		22.17 ± 0.98	−17.28	
Total volume	0.18 ± 0.01	0.999	0.17 ± 0.01	−4.61	0.347	0.17 ± 0.00	−5.55	0.207	0.16 ± 0.01	−11.11	0.201	0.16 ± 0.01	−10.00	0.259	0.16 ± 0.01	−8.33	0.736	0.16 ± 0.01	−8.89	0.820	0.13 ± 0.01	−23.33	0.057
	0.18 ± 0.00		0.17 ± 0.00	−0.94		0.17 ± 0.00	−1.83		0.17 ± 0.00	−5.55		0.17 ± 0.01	−5.88		0.16 ± 0.01	−10.00		0.16 ± 0.01	−7.78		0.15 ± 0.01	−14.83	

MsDex: microspheres loaded with dexamethasone; RNFL: retina nerve fibre layer; GCL: ganglion cell layer; thickness in microns (µm); mean ± SD (SD: standard deviation); *p* < 0.05 statistical significance; *p* < 0.020# statistical significance with Bonferroni correction for multiple comparisons. Cells coloured in grey when right eye (upper cell) showed thinner sectors and/or higher percentage loss compared to left eye (lower cell).

**Figure 6.**
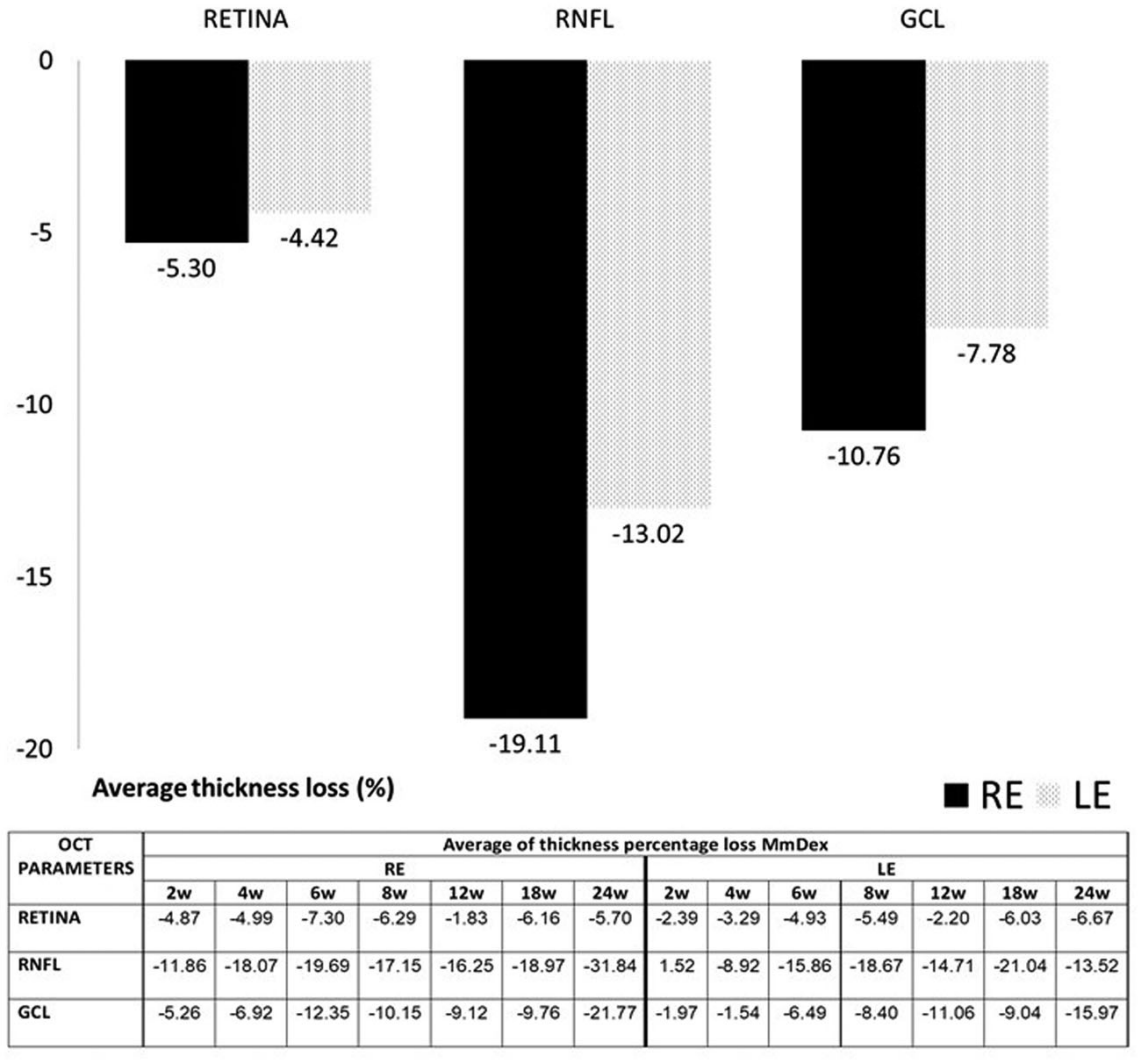
Thickness percentage loss, measured by optical coherence tomography (OCT), in the dexamethasone-loaded microsphere (MsDex) model from baseline to 6 months of follow-up. MsDex: microspheres loaded with dexamethasone; RE: right eye; LE: left eye; w: week; OCT: optical coherence tomography; RNFL: retina nerve fiber layer; GCL: ganglion cell layer; %: percentage.

Figure 6 shows the percentage of thickness loss over time. The RNFL was the parameter that showed the biggest percentage loss in both eyes at every time point and on average. It was followed by the GCL and the retina. Injected REs showed a bigger loss than LEs.

The neuroretinal percentage loss by OCT sectors from the retina, RNFL and GCL was quantified and the loss tendency was analyzed (see Supplementary Figures 1, 2 and 3, respectively). Variability in retina alteration was observed in REs over time, alternating from outer to inner sectors. However, in LEs the outer sectors tended to experience a greater percentage loss in thickness. The same loss trend was found between eyes at Weeks 6 and 24 and on average. This also happened at Week 4 and on average in the RNFL, where the superior-inferior sectors of the vertical axis were the most often altered. In the GCL, the inner sectors showed greater percentage losses in both eyes at every time point; the nasal-temporal sectors of the horizontal axis were the most affected and the inferior sector the least.

**Figure 7.**
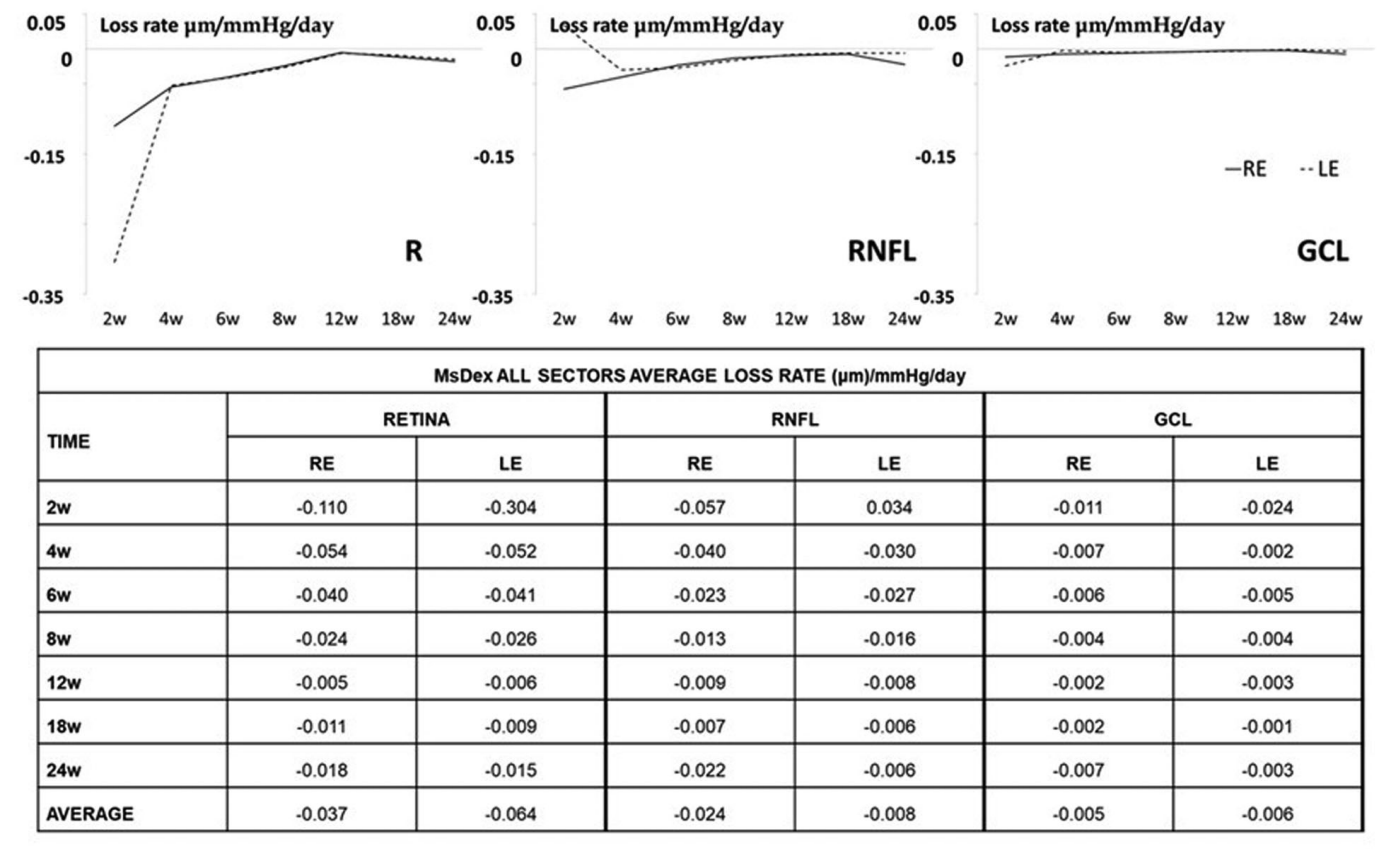
Neuroretinal loss rate, measured by optical coherence tomography (OCT), in the dexamethasone-loaded microsphere (MsDex) model. MsDex: microspheres loaded with dexamethasone; RE: right eye; LE: left eye; w: week; RNFL: retina nerve fiber layer; GCL: ganglion cell layer. Each data is obtained from baseline time.

The average loss rate expressed in microns per mmHg and day extracted from all sectors was also quantified in both eyes and at all stages. The highest levels of thickness loss were found in the retina in the early stages (up to 8 weeks) and in the RNFL in the intermediate and later stages. On average, the highest loss rate was found in the retina, followed by the RNFL and then the GCL. REs showed a higher loss rate in the RNFL but a lower one in the retina than their non-injected contralateral LEs (Figure 7).

#### Electroretinography (ERG):

**Figure 8.**
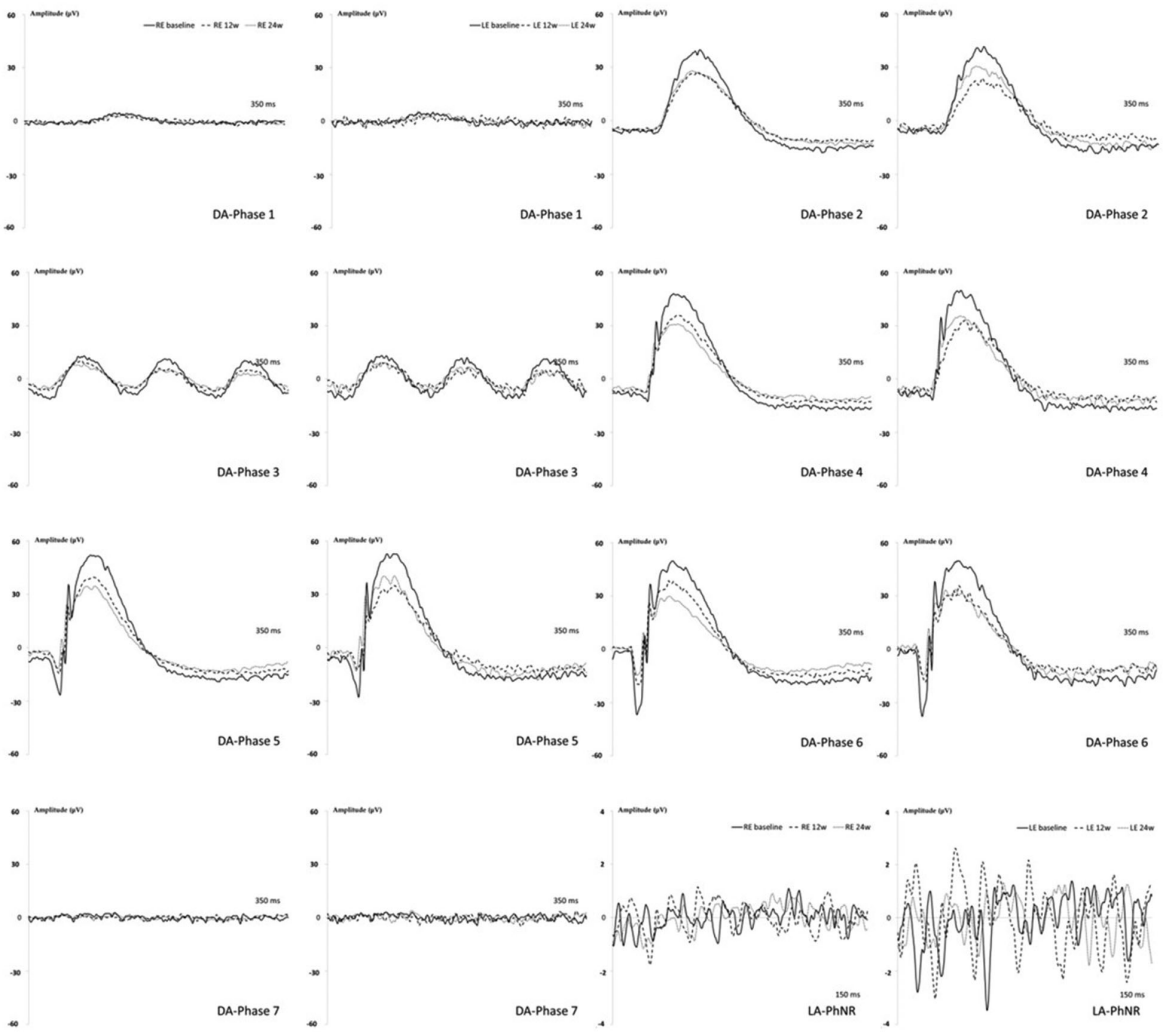
Neuroretina functionality, measured by dark- and light-adapted electroretinography (ERG), in the dexamethasone-loaded microsphere (MsDex) model over 6 months of follow-up. MsDex: microspheres loaded with dexamethasone; RE: right eye; LE: left eye; w: week; DA: dark-adapted; LA: light-adapted; μV: microvolts; ms: milliseconds.

REs showed longer latency and smaller amplitude in all dark-adapted (DA) phases explored over time and the biggest decrease in signal was found from baseline to Week 12. LEs followed a similar pattern but did not keep decreasing at Week 24. The light-adapted (LA) PhNR protocol also showed diminished signal over time, with REs smaller than LEs (see Figure 8).

REs showed statistically decreased amplitudes when compared to LE DA rod response (Phase 4: photoreceptors (*a-wave*): 14.23 ± 9.49 vs 47.38 ± 28.07 μV; *p* = .021) and oscillatory potentials (Phase 7: intermediate cells (*b-wave*): 66.88 ± 26.20 vs 135.13 ± 39.38 μV; *p* = .005), and LA-PhNR (intermediate cells (*b-wave*): 23.05 ± 21.17 vs 75.70 ± 48.78 μV; *p* = .036) at Week 12; and in DA rod response (Phase 1: intermediate cells (*b-wave*): 34.09 ± 22.14 vs 97.88 ± 45.69 μV; *p* = .012) (Phase 2: photoreceptors (*a-wave*): 25.77 ± 16.84 vs 60.87 ± 32.51 μV, *p* = .041) and ganglion cell response LA-PhNR (intermediate cells (*b-wave*): 22.35 ± 12.81 vs 54.40 ± 30.21 μV; *p* = .038) at Week 24.

### Histology

**Figure 9.**
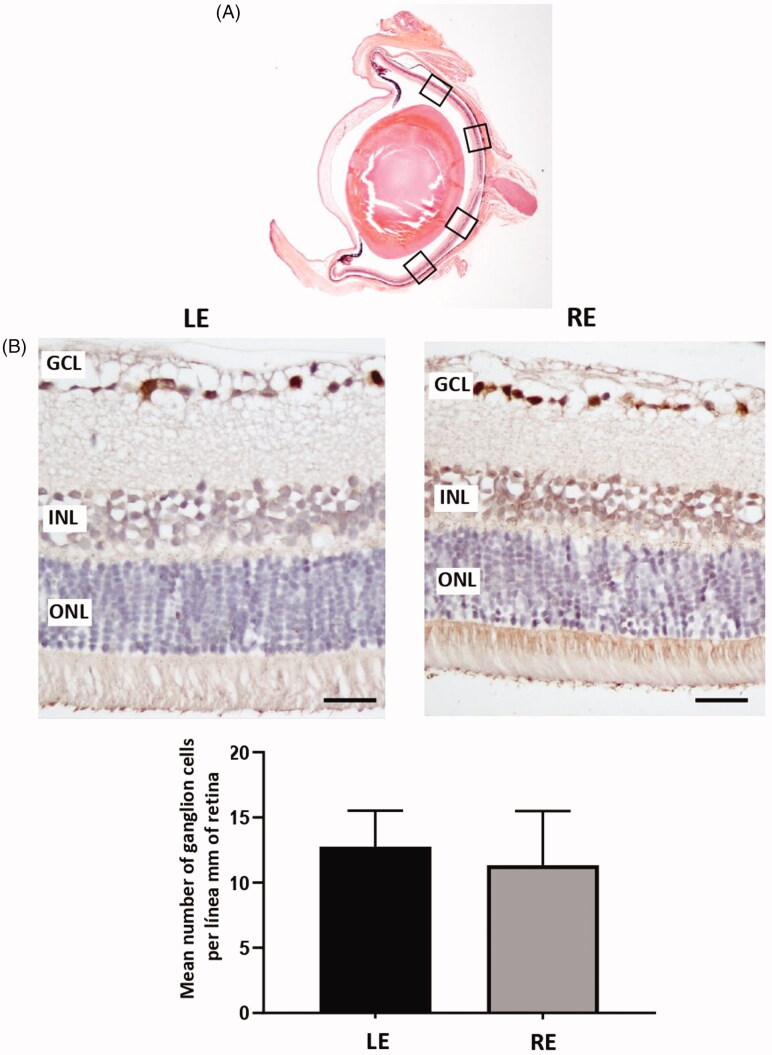
Ganglion cell analysis in glaucomatous eyes. (A) Ganglion cells were counted in four areas (squares) of a radial section of the retina passing through the optic nerve. (B) Two representative images of the retina marked with anti-Brn3a, corresponding to a left control eye (LE) and a right eye (RE) from the same animal injected with dexamethasone microspheres. (B) The mean number of ganglion cells per linear mm of retina did not differ between non-injected eyes and injected eyes. GCL: ganglion cell layer; INL: inner nuclear layer; ONL: outer nuclear layer. Scale bars: 50 μm.

The count of positive Brn3a cells along 2 mm of the retina showed that the mean number of ganglion cells was not significantly different at the study end in LEs when compared with REs injected with dexamethasone microspheres (*p* = .3787) (24 weeks) (Figure 9).

**Figure 10.**
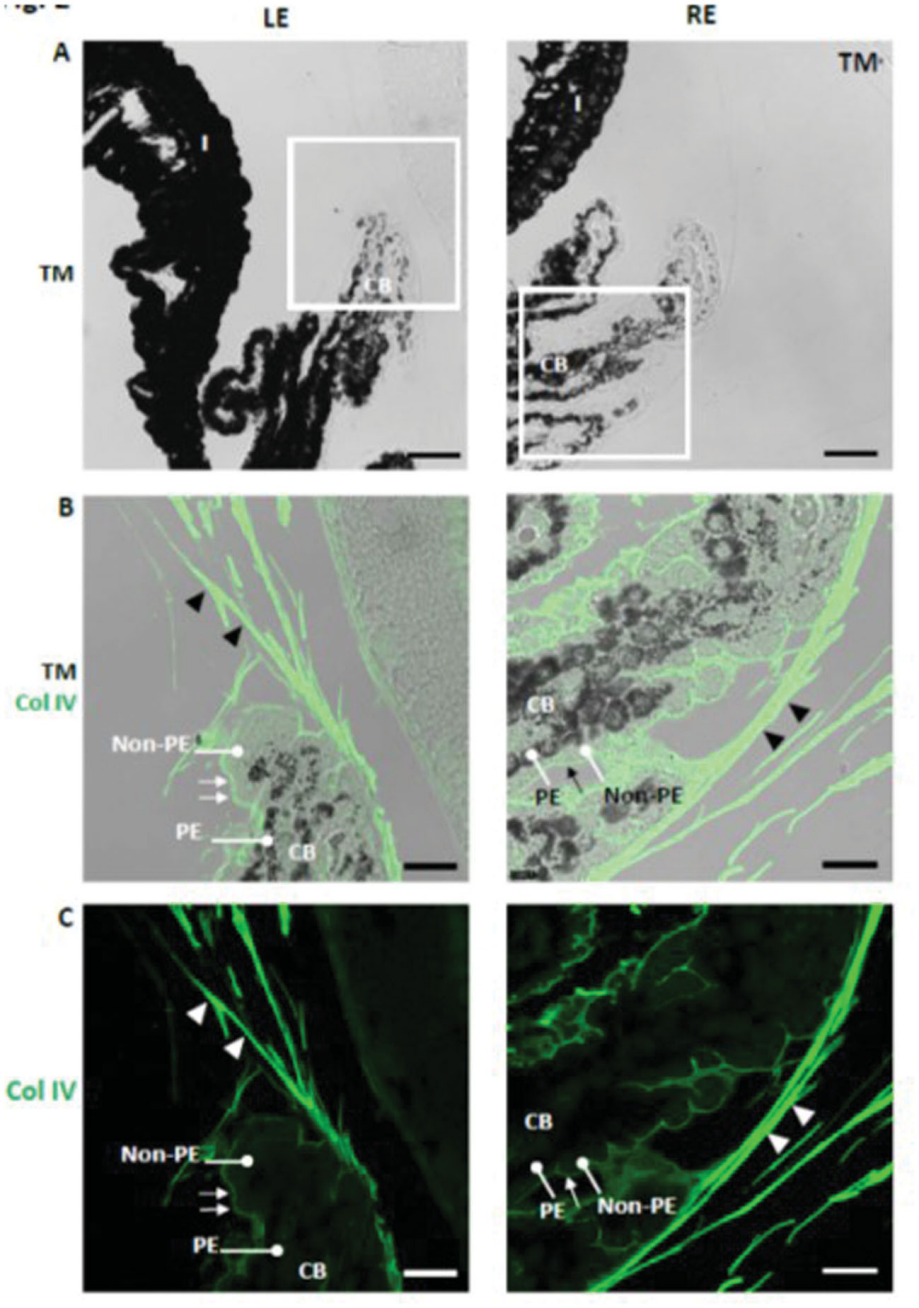
Increased deposition of collagen IV in the zonular fibers (arrowheads) and in the basement membrane of the non-pigmented epithelium (arrows) at the ciliary body in dexamethasone-injected rat eyes. LE: left eye; RE: right eye (injected with dexamethasone-loaded microspheres); CB: ciliary body; non-PE: non-pigmented epithelium; PE: pigmented epithelium; TM: transmission mode. Scale bars: (A) 43.48 μm; (B, C) 18.18 μm.

The mechanism of glucocorticoid-induced aqueous humor outflow obstruction is not well understood but appears to be associated with the accumulation of extracellular matrix material (Overby and Clark, 2015). Greater accumulation of type IV collagen, heparin sulfate proteoglycan, and fibronectin at the trabecular meshwork has been observed in glucocorticoid-induced human glaucoma (Tawara et al., 2008). In our experiment, rat eyes injected with dexamethasone microspheres showed increased deposition of collagen IV in zonular fibers and in the basement membrane of the non-pigmented epithelium at the ciliary body (Figure 10). In contrast with the human ciliary body (Marshall et al., 1992), collagen IV was not detected by immunohistochemistry in the pigmented epithelium basement membrane of the rat ciliary body (Figure 10).

**Figure 11.**
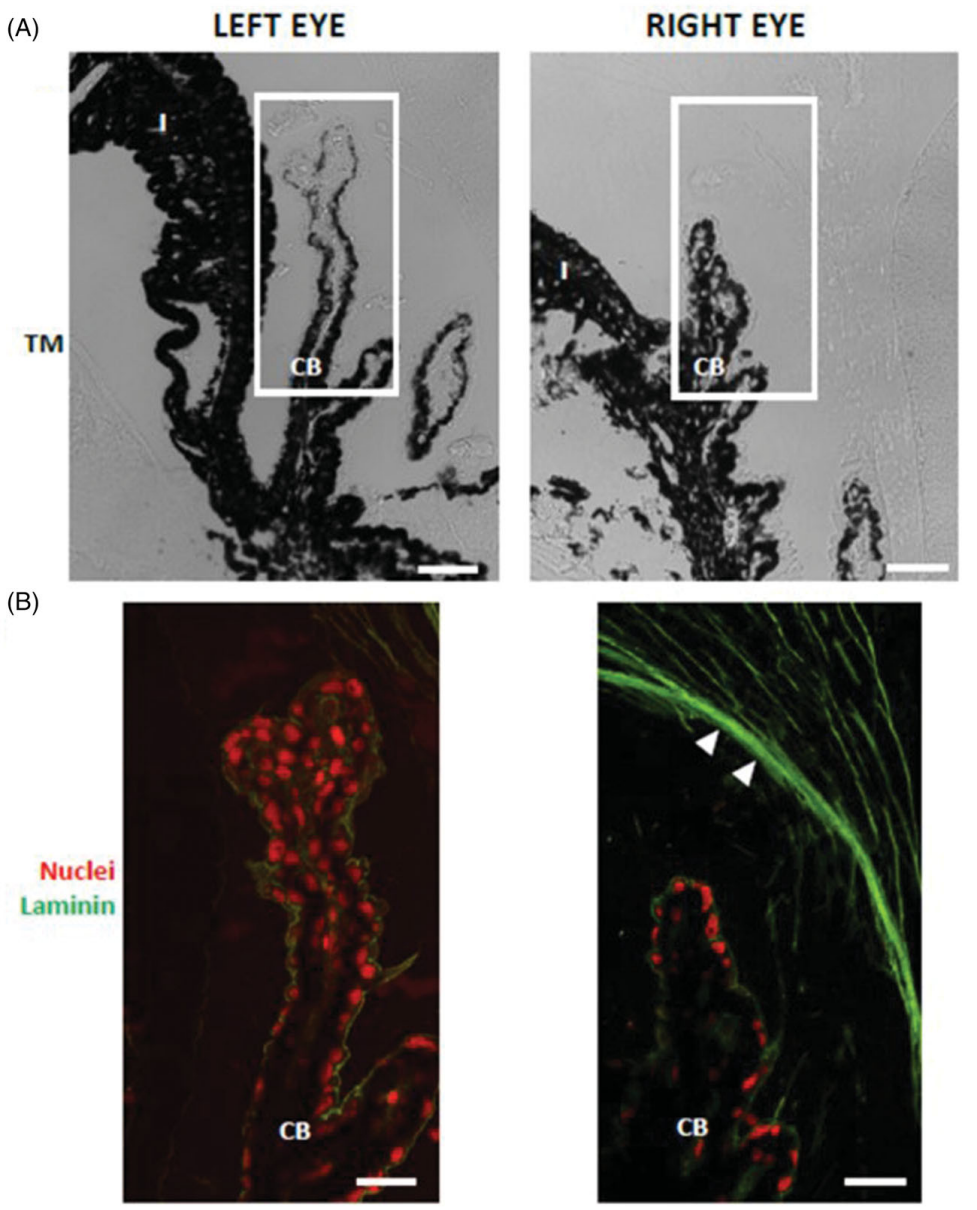
Laminin deposition in the zonular fibers (arrowheads) of dexamethasone-injected rat eyes. The ciliary body in (A) transmission mode (TM) and (B) in fluorescence. RE: right eye (injected with dexamethasone-loaded microspheres); LE: left eye; CB: ciliary body; non-PE: non-pigmented epithelium; PE: pigmented epithelium. Scale bars: (A) 43.48 μm; (B) 20.60 μm.

**Figure 12.**
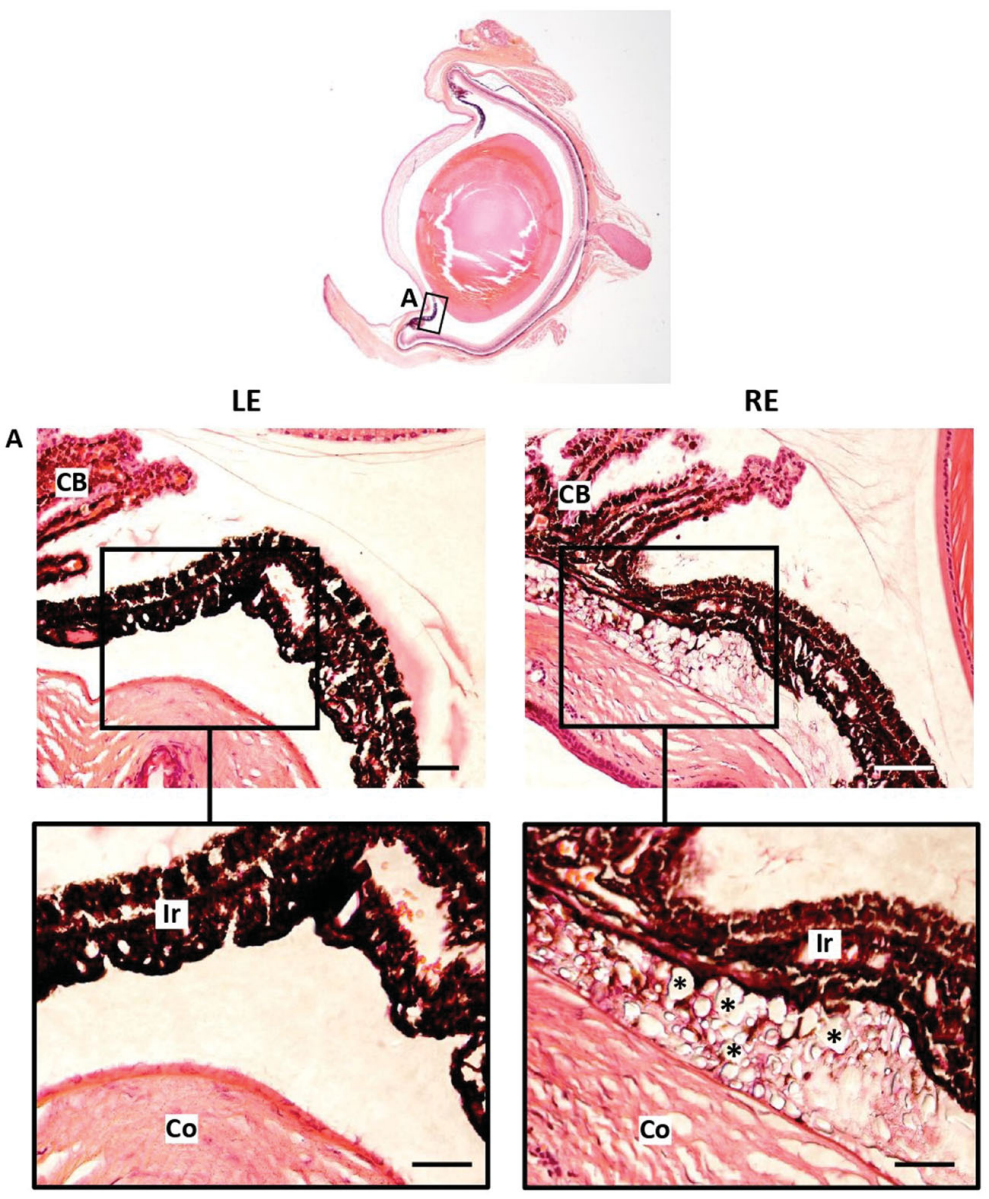
Iridocorneal angle hampering after intracameral injection of dexamethasone-loaded microspheres. A Iridocorneal angle LE Left eye RE Right eye CB Ciliary body Ir Iris Co Cornea Microsphere remnants Scale bars 77 µm (A) 38 µm (inset).

The composition of zonular fibers has been studied using both immunohistochemical and mass spectrometric approaches (Bassnett, 2021). The zonular fibers are composed almost entirely of fibrillin-based microfibrils and three well-characterized microfibril-associated proteins: LTBP-2, MFAP2, and EMILIN-1 (29). Although De Maria et al. (De Maria et al., 2017) identified hundreds of zonular proteins, laminin has not been detected in human and bovine zonular fibers. Similarly, laminin was not detected by immunohistochemistry in left-eye zonular fibers (Figure 11). However, glucocorticoid-induced glaucomatous right eyes showed laminin deposition in zonular fibers (Figure 11). As a hypothesis, our results suggest that collagen IV and laminin deposition at zonular fibers in glucocorticoid-induced hypertensive rat eyes could have disturbed aqueous humor flow and may play a role in the development of glaucoma. According to Figure 12, microspheres aggregates remained in the iridocorneal angle of eyes after intracameral injection of dexamethasone-loaded microspheres 6 months after injection.

## Discussion

A multitude of ocular hypertension models has been developed for the study of glaucoma, including steroid-induced glaucoma. SIG shares similarities with POAG. Patients with POAG present the increased hypertensive response to glucocorticoids, characteristic histological alterations in the trabecular meshwork (Weinstein et al., 1985), and elevated corticosteroid levels in aqueous humor (Kasimov and Aghaeva, 2017). In addition, a relationship between chronic exposure to stress hormones and retinal (O'steen et al., 1987) and neuronal (Vyas et al., 2016) degeneration has been demonstrated. The SIG model is therefore considered a suitable model for the study of glaucomatous neurodegeneration (Agarwal and Agarwal, 2017). Furthermore, the interest in iatrogenic SIG is growing due to the increase in ophthalmologic pathologies associated with aging that require treatment with glucocorticoids (GC) (Phulke et al., 2017), so knowledge of its pathophysiology, along with better-characterized models that reproduce it, are in demand.

**The ideal glaucoma model** (Biswas and Wan, 2019) is one that meets the following criteria: (1) easily induced, (2) biologically plausible and with minimal side effects, (3) reproducible, (4) predictable in onset and clinical course and, finally and most importantly, (5) simulates the pathophysiology of glaucoma, i.e. glaucomatous damage with decreased axonal fibers and RGC and increased cup/disk-optic associated with increased IOP. However, current models present complications or limitations (very high hypertension in episcleral vein sclerosis or cauterization, or corneal edema and synechiae, in non-biodegradable microspheres) that hinder longitudinal *in vivo* monitoring and disease dynamics, and it is difficult to identify those time windows where early/intermediate/late intervention would be useful (Kowal et al., 2021). Our model meets all the above requirements and minimizes complications or limitations as much as possible.

**The microsphere model** is a consequence of the physical blockage of the outflow of aqueous humor by clogging the trabecular meshwork. Its technical requirements are less demanding than those of other models such as the episcleral model (Morrison et al., 2015). The non-biodegradable microsphere model (Biswas and Wan, 2019) ranks second (with respect to the other hypertensive models) in terms of generating IOP elevation with RGC loss and appears to be the only one that demonstrates a negative trend between OHT duration and RGC loss. However, it requires reinjections to maintain an elevated IOP. Our group created a glaucoma model by administering biodegradable PLGA microspheres into the anterior chamber of rat eyes to generate ocular hypertension (Garcia-Herranz et al., 2021). A subsequent 6-month study (Rodrigo et al., 2021) using 7 injections demonstrated the reproducibility of the new model and produced functional and structural damage while maintaining superb ocular tolerance that enabled serial *in vivo* functional and structural testing with reliable results.

Over the past 30 years, lactic and glycolic acid copolymers (PLGA) have been implemented in biomedicine for diagnosis and treatment. This biomaterial is approved by the U.S. Food and Drug Administration (FDA) and the European Medicines Agency for use in human beings due to its biocompatibility and biodegradability (Martins et al., 2018). PLA, PGA, and PLGA polymers have been used to create controlled drug-delivery systems. An intraocular PLGA implant loaded with dexamethasone is already available in ophthalmological clinical care (Ozurdex®) (Chang-Lin et al., 2011). In addition, it is well known that PLGA Ms can encapsulate and subsequently sustainedly release corticosteroids such as dexamethasone for several months (Barcia et al., 2009; Dawes et al., 2012; Barbosa-Alfaro et al., 2021). Depending on Ms particle size, among other technical parameters, it is common to find an initial rapid release of the active compound followed by one or several low-rate release phases. In our case, as a second injection of the formulation was performed in the *in vitro* release study to mimic the *in vivo* administration, the two release profiles overlapped.It has already been shown in many previous works that the degradation of PLGA matrices is a homogeneous degradation, which, unlike the heterogeneous degradation of polymers in the matrices, takes place in the entire polymer chain at the same time. In aqueous media, although partially aggregated, PLGA microspheres maintain a spherical external appearance for weeks, despite the continuous degradation by hydrolysis of PLGA until a moment comes when the structures collapse, becoming polymeric aggregates without definite shape (Checa-Casalengua et al., 2011; Barbosa-Alfaro et al., 2021). Although a similar behavior can be assumed *in vivo*, it is very difficult to establish the exact behavior of these PLGA microspheres once injected into the anterior chamber. However, in this context, the physical blockage can be explained by the presence of both the pellet (limiting the aqueous humor outflow) and the remaining isolated PLGA material that infiltrates through the trabecular pores, that according to literature, offer porous of around 25–27 µm in the uveal meshwork of the TM, being significantly narrower in the corneoscleral meshwork of the TM (2–15 µm) (Abu-Hassan et al., 2014). In fact, remnants of microspheres were observed at the iridocorneal angle of eyes after intracameral injection of dexamethasone-loaded microspheres according to (Figure 12).

Those models that require frequent reinterventions produce more complications. To decrease intervention in the animal, and thanks to the sustained release of dexamethasone from MsPLGA (Rodríguez Villanueva et al., 2016), the present model was developed in which only 2 injections of the MsPLGA formulation loaded with dexamethasone were necessary to obtain a slow and sustained hypertensive curve that produced functional and structural damage over 6 months. Those models with rapid IOP elevations are the least representative of POAG, and the key to success in translational studies is the choice of a model that closely represents the human disease (Agarwal and Agarwal, 2017) (Table 2). In our case, it was decided to perform 2 ocular injections to obtain an initial mechanical microsphere blockade and a progressive alteration of the trabecular meshwork utilizing a pharmacological mechanism secondary to the sustained release of dexamethasone. This was performed to obtain a slow IOP rise curve that resembled human glaucoma, in addition to improving animal welfare, reducing costs, and reducing the risk of other complications such as infection or ocular toxicity due to high concentrations of PLGA (Park, 2017; Zhao et al., 2017).

**Table 2.** IOP-induced glaucoma models.

	Postrabecular			Trabecular		Pretrabecular			
Glaucoma models by OHT	Intermitent and transient (Crowston et al. 2015)	Sclerosis epiescleral veins (J C Morrison et al. 1997)	Cauterization epiescleral veins (Garcia-Valenzuela et al. 1995)	Laser photo-coagulation (Levkovitch-Verbin et al. 2002)	Transduction of TM with genetic (Kersey and (Shepard et al. 2010)	Intracameral administration of viscous agents Benozzi et al. 2002)	Microspheres non-biodegradable (Samsel et al. 2011; Urcola, Hernández, and Vecino 2006)	Microspheres biodegradable	Microspheres PLGA-DEX
Induction	Surgery.	Surgery.	Cauterization.	Laser equipment needed.	Gene edition.	Corneal puncture.	Corneal puncture.	Corneal puncture.	Corneal puncture.
	Challenging	Challenging	Easy	Challenging	Challenging	Easy	Easy	Easy	Easy
Interventionism	Loop/cannulation	Injection.	Burn.	Laser.	Modified animal genetics.	Injections.	Injections.	Injections.	Bi-injection
	Multiple	Single/ Multiple	Single	Single	–	Multiple	Multiple	Multiple	
Side effects	Ocular ischemia	Cornea cloud, cataract	Ocular inflammation, vasocongection	Ocular inflammation. Unintended ocular injuries.	Gen edition. Ocular inflammation	No described	Corneal edema, sinechiaes, ischemia	Periferal corneal leucoma	Periferal corneal leucoma, cataract
Iop increase	Spikes.	Spectrum of IOP.	Spikes.	Spikes.	Progressive	Spikes	Progressive and sustained	Progressive and sustained	Progressive and sustained
	Acute OHT	Rapid and sustained	Rapid and sustained	Transient elevation	–				
Neuroretinal degeneration	7–52% RGC loss	10–100%ON axon loss	4% RGC loss per week	50–70% ON axon loss	13% ON axon loss	40% RGC loss	23-6%RGC loss	−9.81µm RNFL (OCT)	−19.11µm RNFL (OCT)
								−12.88µm GCL (OCT)	−10.76µm GCL (OCT)
Duration	Short. 30 min–8h.	Multiple → 26w.	1 cauterization →16w.	1 laser →1 year.	6w	1 injection →1 w. Weekly up to 10w.	2 injections → 8w. weekly up to 30w.	7 injections → 24w	2 injections → 24 w.

OHT: ocular hypertension; IOP: intraocular pressure; TM: trabecular meshwork; ON: optic nerve; RGC: retinal ganglion cell; RNFL: retinal nerve fiber layer; GCL: ganglion cell layer; OCT: optical coherence tomography; w: week; %: percentage; min: minutes; h: hours; µm: microns (22) (Pang and Clark 2020).

**Focusing on SIG models** performed in rodents (Rybkin et al., 2017), most use mice and the effect of glucocorticoids on OHT and ocular function and structure has been extensively studied in them (Overby et al., 2014; Zode et al., 2014; Faralli et al., 2018; Patel et al., 2018; Li et al., 2019; Patel et al., 2019). Topical administration produces the greatest OHT with retinal dysfunction and optic neuropathy but has the disadvantage of requiring daily administration. Subconjunctival administration reduced outflow but required repeated injections to maintain elevated IOP (Li et al., 2019). Systemic administration with a minipump allows dexamethasone release for several weeks without intervention but produced a high rate of deaths (Overby and Clark, 2015) (Table 3). However, few SIG studies in rats have suggested differences with mice. Very few studies have analyzed the impact on IOP (Sawaguchi et al., 2005; Shinzato et al., 2007; Miyara et al., 2008; Razali et al., 2016; Sato et al., 2016), as well as the structural changes in the trabecular meshwork (Sawaguchi et al., 2005; Shinzato et al., 2007; Razali et al., 2016) and retinal structure (Miyara et al., 2008; Razali et al., 2016). To our knowledge, there are none that explore the effects of GCs on the structure using OCT or retinal functionality and all are performed by daily topical administration with a maximum follow-up of 8 weeks.

**Table 3.** Steroid-induced glaucoma models.

SIG models	Systemic (Whitlock et al. 2010)	Periocular (Wang et al. 2018; Li et al. 2019)	Anterior chamber	Topical (Shinzato et al. 2007; Sato et al. 2016)
Induction	Surgery	Subconjuntival injection	Corneal puncture	Drops, ointment
	Subcutaneous minipump	Nanoparticles PLGA-DEX	Microspheres PLGA-DEX	
	Challenging	Easy	Easy	Easy
Interventionism	1	3	2	Daily
Side effects	Systemic, body weight loss, leucopenia, infection, death	Prolonged duration of action, non-predictability, no visualization, inability to withdraw	Periferal corneal leucoma, cataract	Bodyweight loss
IOP increase	Mild and progressive	Progressive and sustained	Progressive and sustained	Progressive and sustained
Neuroretinal degeneration	-No data	-No data	−19.11 µm RNFL (OCT)	29% axon loss (at 15w)
			−10.76 µm GCL (OCT)	
Duration	4w	8w	24w	8w

SIG: Steroid induced glaucoma; IOP: intraocular pressure; RNFL: retinal nerve fiber layer; GCL: ganglion cell layer; OCT: optical coherence tomography; w: week; %: percentage; µm: microns.

**Our SIG model** reduces the repeated interventionism of daily topical application and the potential negative side effects of systemic administration or periocular administration (Li et al., 2019), which is considered the most dangerous because of its prolonged duration of action, non-predictability of response, and the inability to withdraw it in the event of hyper-corticosteroid-responsiveness, in addition to the impossibility of visualization (Phulke et al., 2017). In contrast, anterior chamber administration allows visualization of the Ms and control of the model by modulating the injections. Moreover, if required by the researcher, repeated injections can modulate and control the hypertensive curve. In addition, our MsPLGA-Dex SIG model was well tolerated, as no animal developed an infection and only one developed cataract, even though it is known that chronic exposure to GC increases the risk of developing them (James, 2007; Migita et al., 2013).

Our model produced a slightly earlier **IOP** increase (1 vs 2–3 weeks), possibly due to the synergy of the mechanical blockage of PLGA microspheres combined with the pharmacological action of the presumed presence of dexamethasone in the area, and reached OHT later (5 vs 2–4 weeks) than previous studies in rats (Sawaguchi et al., 2005; Miyara et al., 2008). In fact, when comparing the IOP obtained at week 1 in animals induced with non-loaded microspheres (data obtained from our previous work (Rodrigo et al., 2021), with respect to animals with microspheres loaded with dexamethasone (13.98 ± 3.08 mmHg vs. 15.3 9 ± 2.93 mmHg) since the groups were comparable because they received only one injection at baseline (same size of Ms and volume), the difference in IOP was probably due to the dexamethasone released at that time.

In addition, the **corticosteroid response** was analyzed, being to our knowledge the first study to do so. OHT response to corticosteroids varies between patients and SIG models, but typically there is an IOP increase after the third week of exposure to corticosteroids. Between 30 and 40% of humans respond to corticosteroid with an IOP increase of 6–15 mmHg (medium responders); 4–6% respond with an increase of more than 15 mmHg (high responders); and in 1–3% of patients, IOP remains high even after ceasing exposure to steroids (Clark, 1995; Kersey and Broadway, 2006; Clark and Wordinger, 2009). The percentage of OHT corticosteroid response varies in other animals (50% of primates, 80% of rats, and 100% of cows) (Danias et al., 2011). In our rat model, the maximum percentage of hypertensive animals was similar to previous studies (80–90%) (Razali et al., 2015) and mostly medium corticosteroid responsiveness was found, as in most humans.

An increase in IOP was also detected in the **contralateral eye** at 8 weeks (later than other authors) (Rybkin et al., 2017). In this regard, in a mouse SIG model using the periocular injection of PLGA-Dex nanoparticles (Li et al., 2019), an increase in IOP was detected in the contralateral eye, and possible perilymphatic communication was hypothesized, but could not be fully explained (Zode et al., 2014; Patel et al., 2017; 2018). Other reasons could be direct systemic vascular communication and periocular lipophilic deposition with the subsequent systemic release of very low and chronic levels that end up in the aqueous humor as plasma ultrafiltrate and alter the genomics and functionality of the trabecular meshwork (see section Hypothesis of the effect of glucocorticoids in the glaucoma model and in the future). In this regard, human studies have shown elevated levels of GC in the aqueous humor (Chang-Lin et al., 2011; Kasimov and Aghaeva, 2017). However, all of the above are hypotheses and it would be beneficial to measure the levels of dexamethasone in the aqueous humor of both eyes in our model to corroborate this. Although our histological results found no increased laminin deposition in the contralateral eye, it nevertheless suffered a progressive increase in IOP, meaning there are still unknowns to be resolved. Our model would be the first to analyze such an increase in IOP, even reaching OHT in the contralateral eye over a long study period.

**Neuroretinal degeneration** was noninvasively assessed longitudinally and *in vivo* using OCT, allowing for longer tracking than in previous studies. Bilateral thickness loss was detected; it was greater in the injected right eye and was mainly in the RNFL (superior-inferior axis sectors) (Guo et al., 2010), followed by the GCL (nasal-temporal sectors) (Salinas-Navarro et al., 2009). The reduction in the loss rate over time was probably due to the floor effect. The bilateral loss meant that no statistically significant differences in ganglion cell counts at 24 weeks were found in histological studies. However, fewer RGCs were counted than in our previous model with unloaded PLGA microspheres (Garcia-Herranz et al., 2021). RNFL > GCL > R involvement may reflect the anterograde neurodegenerative pathway, as the same trend of topographic loss in depth (from inner to outer layers) was maintained throughout the 24 weeks. Likewise, the involvement of the contralateral left eye could be due to the increase in IOP (mentioned above) and/or the dissemination and activation of inflammatory factors due to transsynaptic degeneration (Sapienza et al., 2016; de Hoz et al., 2018; Lawlor et al., 2018). Immune activation has been detected in histological studies of glaucoma (Ramirez et al., 2017). In our study, OCT showed dynamic fluctuations over time and a topographic variability in retinal thickness that could reflect immune activation and infiltration in waves (Ramirez et al., 2017). It cannot be ruled out that the observed fluctuations may be due in part to the variability of the device. However, the fact that follows up technology was used (which allows measurement at the same exact points in each re-evaluation) and that the fluctuations were observed at the same study times as in previous work performed in healthy (Rodrigo et al., 2020) and glaucomatous (Rodrigo et al., 2021) animals with OCT, as well as in histological studies that related the fluctuations to immune infiltration; makes the authors of the study consider it unlikely to be a failure or variability of the technique and they do consider it to be a sign of reproducibility in the measurement, in agreement with the recognition of the influence of immunity in glaucoma (Geyer and Levo, 2020).

We found a greater thickness in the inner sectors of the R in the contralateral left eye, which could reflect a possible infiltration from the central vessels and the optic nerve due to dissemination from the contralateral eye. This coincides with an increase in RNFL observed at week 2 in the loss rate. On the other hand, the loss of inner sectors of the GCL (Davis et al., 2016) was constant in both eyes, suggesting initial damage close to the optic nerve (Howell et al., 2007). Possible early immune infiltration of the retina in the induced right eye resulted in lower loss rates compared to the contralateral left eye. In the early weeks of the study (weeks 2–4), the animal's eye is still growing, which was quantified as a higher rate of retinal thickness loss in the uninduced left eye (Rodrigo et al., 2020). Possible immune infiltration of the retina due to noxa (Sapienza et al., 2016; de Hoz et al., 2018; Rodrigo et al., 2021) was quantified in the right eye as a lower rate of loss and thus suggests being an underestimate measured with OCT.

ERG scans revealed a loss of neuroretinal functionality over time. The injected right eye always showed lower functional signal under both scotopic and photopic conditions; however, the left eye also suffered moderate functional limitation as the signal enhancement caused by neuromodulation described in healthy animals 2–3 months old was not detected (Chaychi et al., 2015; Nadal-Nicolás et al., 2018; Rodrigo et al., 2020). This suggests that the hypertensive noxa in the left eye, lasting only 4 weeks, exerted a damaging/limiting influence. In this regard, in glaucoma, relatively few changes have been described in scotopic ERG, although there have been more significant alterations with pSTR and oscillatory potentials (Bach and Poloschek, 2013; Park et al., 2018). In our study, the phases with lower intensities (and therefore close to the pSTR), as well as oscillatory potentials, were the most affected (Figure 8). Only one study with SIG in rodents (C57bL/6 mice) analyzed retinal function using pattern ERG (specific for RGC), at 20 weeks finding a 55% loss of signal under photopic conditions (Zode et al., 2014). Our study seems to be the first to analyze the effect of SIG on retinal function in rats, and for a longer time (24 weeks), detecting a decrease in the response under photopic conditions of close to 50% of the PhNR at week 24, as well as signal deterioration under scotopic conditions from week 12 onwards.

**The hypothesis of the effect of glucocorticoids in the glaucoma model and in the future**. GCs have distinct effects on a multitude of cells, acting as activating or repressor gene modulators (Spies et al., 2011; Cain and Cidlowski, 2017). GCs influence trabecular meshwork cells resulting in cell death. This produces an increase in IOP that triggers an immune response (Chen et al., 2018). Recently, Glaucoma has been named an autoimmune disease (Geyer and Levo, 2020) and, it has also been shown that GCs play a role in activating innate immunity (Cain and Cidlowski, 2017). It has been hypothesized that corresponding to physiological levels of GCs, macrophages (cells of the trabecular meshwork that have phagocytic action, and microglia resident in the retina) are sensitized for rapid detection of damage and, in the inflammatory state, restrain inflammation (Spies et al., 2011). In our case, the chronic release of GC in the order of μg per day could, to some extent, resemble the pathophysiology of glaucoma, where very low concentrations of GC in a chronic subinflammatory state could cause genomic and subsequently cellular/tissue/organ level changes. In this regard, the modified release of prednisone at low doses over a long period of time seems to contribute to chronic changes made to the genome (Spies et al., 2011). Cases of rebellious SIG that end up requiring surgery could be a consequence of already irreversible genomic changes in which conventional treatment is insufficient. On the other hand, the greater the exposure to chronic GC, the greater the inflammatory response (Vyas et al., 2016), and greater inflammation has been associated with greater neuronal damage (González et al., 2014; Noailles et al., 2014).

In this sense, the initial slight increase in IOP due to mechanical blockade by the Ms could trigger the immune response, and corticosteroids could further activate it. In addition, the changes that GCs cause in the trabecular meshwork would further limit the outflow of aqueous humor, reaching OHT levels and perpetuating the damage to the neuroretinal degenerative cascade. However, this mechanistic theory of aqueous outflow limitation may not be so simple, since the bioinformatic analysis of the anterior segment has recently shown that dexamethasone-induced glaucoma involves genes related to different metabolic pathways, tyrosine or cholesterol homeostasis (Song et al., 2011; Zhang et al., 2019). By virtue of its capacity to facilitate longitudinal study over time, our model would allow us to analyze possible connections between the pathways involved (with genomic/proteomic studies) and, therefore, to develop treatments depending on the clinical situation of the patient. Likewise, long-term formulations could be tested, since SIG treatments have only been able to prove their short-term effects up to a maximum of 40 days (Ganekal et al., 2014; Borrás et al., 2016; Razali et al., 2016).

The main **limitations** of this study could also be its potential strengths. While the rigidity of the trabecular meshwork or outflow of aqueous humor has not been assessed by noninvasive techniques such as OCT, neither have proteomic, molecular or genetic studies been performed, or possible differences by sex evaluated. In our opinion, this study would be the first that would make it possible to perform the long-term assessment in a model that more closely resembles the human condition. In future research, it would be ideal to develop a model with minimal interventionism on the animal, for example with a single intervention/injection. Furthermore, it cannot be ruled out that the use of dexamethasone, used as an inducer of OHT, could partly counteract the proinflammatory effect secondary to the increase in IOP. It seems desirable to develop technologies to obtain the same effect (gradual IOP increase with progressive neuroretinal degeneration) without altering immunity.

**In conclusion**, this work explores the usefulness of drug delivery systems, not to treat pathology but to induce it. We have developed a biodegradable, biocompatible microsphere-based delivery system that creates a chronic glucocorticoid- and mechanically induced glaucoma model that mimics human steroid-induced glaucoma produced by increased IOP and retina and optic nerve degeneration. This model could be modified by increasing the number or injections, the quantity of dexamethasone loaded or even the rate of degradation, although in our case only two injections were needed to obtain the outcomes at 24 weeks. As far as we know, this is the first study that would allow the analysis of the earliest and most chronic pathophysiological changes occurring in SIG.

## Supplementary Material

Supplemental Material
