# Supplementary figures and images for: Long-term corticosteroid-induced chronic glaucoma model produced by intracameral injection of dexamethasone-loaded PLGA microspheres
Source: Drug Deliv. 2021 Nov 12;28(1):2427–46. doi: 10.1080/10717544.2021.1998245 (PMC8592597; doi:10.1080/10717544.2021.1998245)

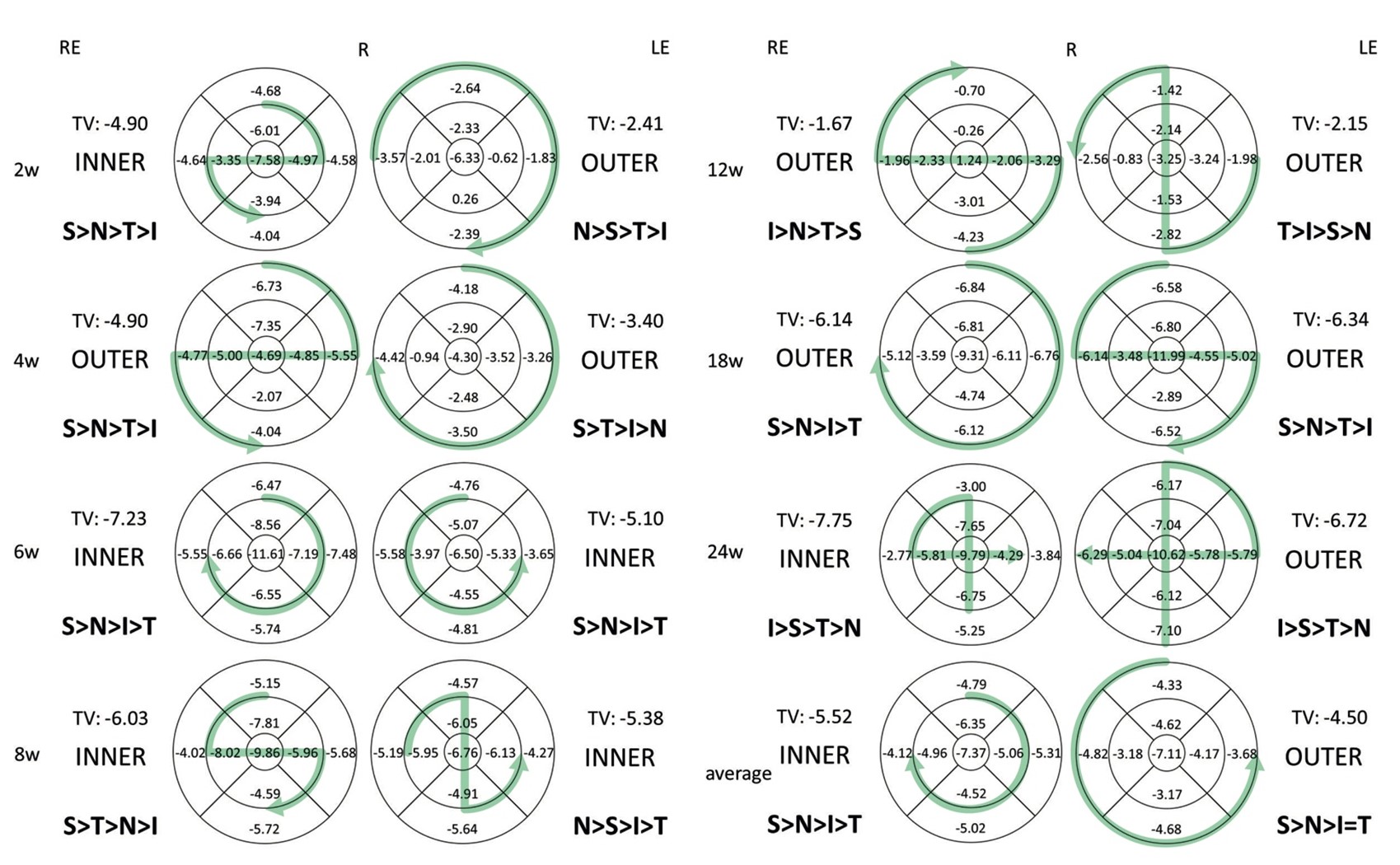

Supplement: Supplemental Material [file IDRD_A_1998245_SM1325.zip › Supplementary Figure 1 previously Fig 7.jpg]

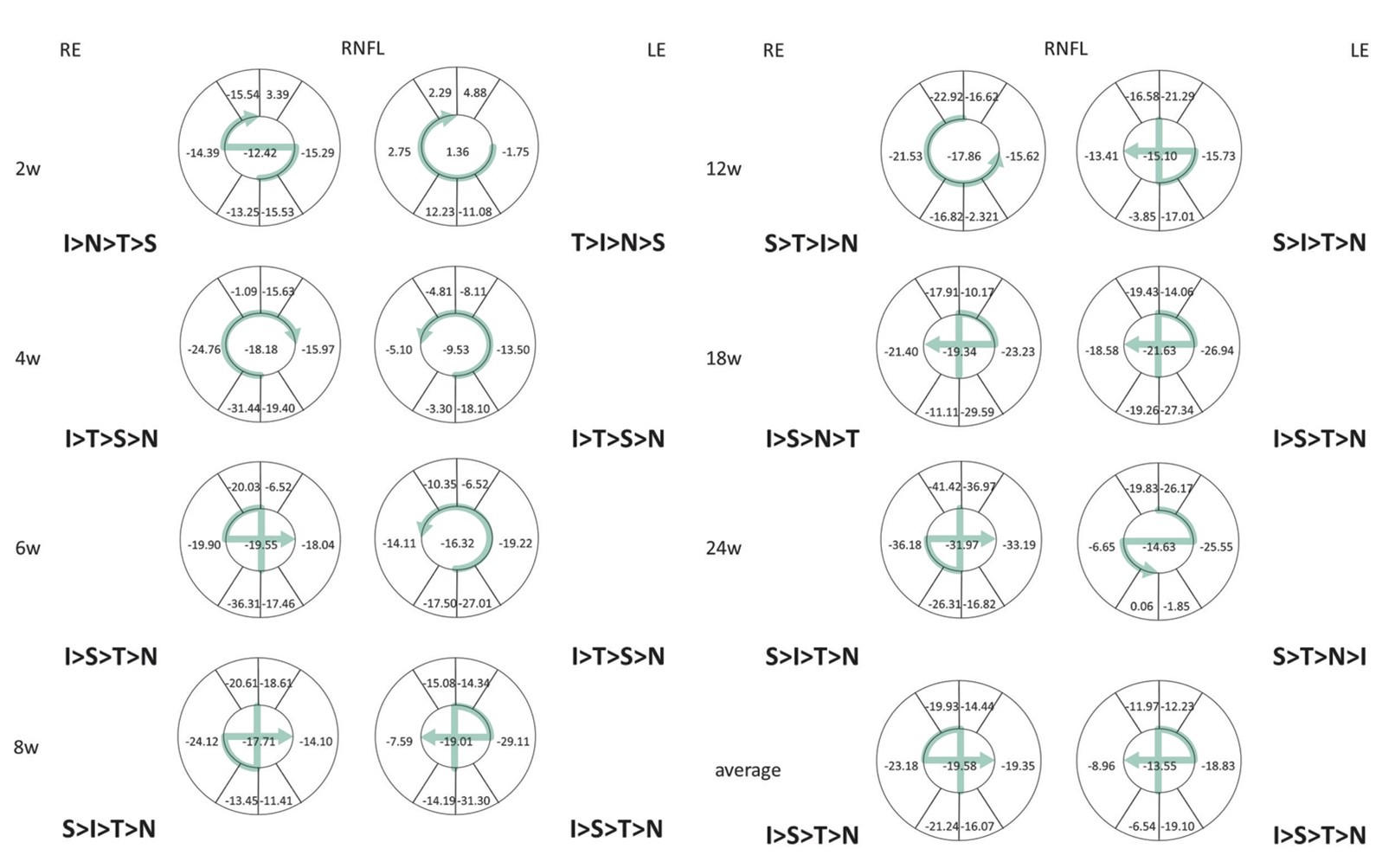

Supplement: Supplemental Material [file IDRD_A_1998245_SM1325.zip › Supplementary Figure 2 previously Fig 8.jpg]

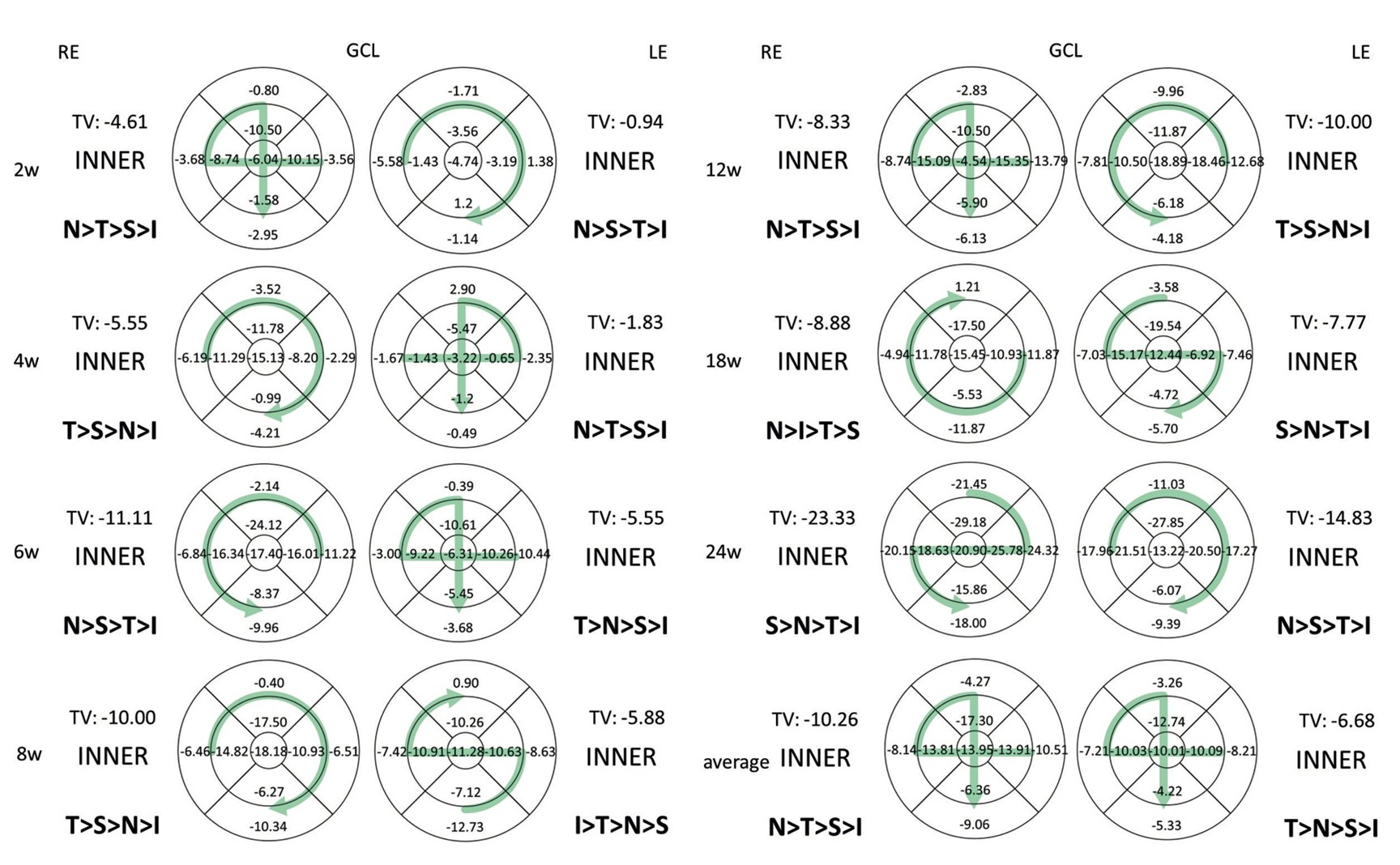

Supplement: Supplemental Material [file IDRD_A_1998245_SM1325.zip › Supplementary Figure 3 previously Fig 9.jpg]
